# Supplementary material for: Measuring health-related quality of life in the general population and Roma communities in Romania: study protocol for two cross-sectional studies
Source: BMJ Open. 2019 Aug 18;9(8):e029067. doi: 10.1136/bmjopen-2019-029067 (PMC6701682; doi:10.1136/bmjopen-2019-029067)
Supplement: Author's manuscript [file bmjopen-2019-029067.draft_revisions.pdf]

BMJ Open

BMJ Open is committed to open peer review. As part of this commitment we make the peer review history of every article we publish publicly available.

When an article is published we post the peer reviewers' comments and the authors' responses online. We also post the versions of the paper that were used during peer review. These are the versions that the peer review comments apply to.

The versions of the paper that follow are the versions that were submitted during the peer review process. They are not the versions of record or the final published versions. They should not be cited or distributed as the published version of this manuscript.

BMJ Open is an open access journal and the full, final, typeset and author-corrected version of record of the manuscript is available on our site with no access controls, subscription charges or pay-per-view fees (http://bmjopen.bmj.com).

If you have any questions on BMJ Open's open peer review process please email info.bmjopen@bmj.com

BMJ Open

Surveys on health-related quality of life in Romania: study protocols

Journal:	*BMJ Open*
Manuscript ID	bmjopen-2019-029067
Article Type:	Protocol
Date Submitted by the Author:	10-Jan-2019

Complete List of Authors:	Olariu, Elena; Newcastle University, Institute of Health and Society<br>Paveliu, Sorin; Universitatea Titu Maiorescu Facultatea de Medicina, Department of Pharmacology and Pharmaeconomics<br>Baican, Eugen; Universitatea Babes-Bolyai, Department of Social Work<br>Oluboyede , Yemi; Newcastle University, Health Economics Group, Institute of Health and Society<br>Vale, Luke; Newcastle University, Health Economics Group, Institute of Health and Society<br>NICULESCU-ARON, ILEANA GABRIELA ; Academia de Studii Economice din Bucuresti, Department of Statistics and Econometrics, Faculty of Economic Cybernetics, Statistics and Informatics

Keywords:	HEALTH ECONOMICS, Health policy < HEALTH SERVICES ADMINISTRATION & MANAGEMENT, Quality in health care < HEALTH SERVICES ADMINISTRATION & MANAGEMENT, PUBLIC HEALTH, STATISTICS & RESEARCH METHODS

SCHOLARONE™
Manuscripts

Surveys on health-related quality of life in Romania: study protocols

Elena Olariu[1] , Sorin Paveliu[2]*, Eugen Baican[3], Yemi Oluboyede[1], Luke Vale[1], Ileana Gabriela Niculescu-Aron[4]

[1] Health Economics Group, Institute of Health and Society, Newcastle University, Newcastle upon Tyne, United Kingdom

[2] Department of Pharmacology and Pharmaeconomics, Faculty of General Medicine, Titu Maiorescu University, Bucharest, Romania

[3] Department of Social Work, Babes-Bolyai University, Cluj-Napoca, Romania

[4] Department of Statistics and Econometrics, Faculty of Economic Cybernetics, Statistics and Informatics, Bucharest University of Economic Studies, Bucharest, Romania

* Corresponding author

Address for correspondence: Sorin Paveliu MD, PhD, Faculty of General Medicine, Titu Maiorescu University, 67A Gheorghe Petrascu Str., district 3, Bucharest, code 031593, Romania. Email: sorinpaveliu@yahoo.com. Telephone: 0040726.200.627.

Word count: 3,968 words

For peer review only - http://bmjopen.bmj.com/site/about/guidelines.xhtml

Abstract

Introduction

Lately, the importance of health-related quality of life (HRQoL) has increased and many healthcare authorities recommend the use of measures that account for both mortality and morbidity. This study will determine, for the first time in Romania, the value sets for EQ-5D-3L and EQ-5D-5L questionnaires and their population norms (study one). It also aims to compare the HRQoL (measured with EQ-5D-5L) of the Roma communities in Romania with that of the general population (study two).

Methods&analysis

Cross-sectional studies of face-to-face interviews conducted in representative samples of the Romanian general population and Romanian Roma communities. 1,614 non-institutionalized adults older than 18 will be interviewed using a computer-assisted interview for study one. Participants will complete EQ-5D-3L and 5L, 13 composite time-trade-off tasks (cTTO), seven discrete choice experiment questions (DCE) and sociodemographic questions. For study two, 606 non-institutionalized self-identified Roma older than 18 will be interviewed using a pencil-and-paper interview. Participants will complete EQ-5D-5L and the same sociodemographic questions as for study one.

The 3L value set will be estimated based on cTTO data using econometric models and the 5L value set using both cTTO and DCE data. Population norms will be reported by age and gender. The odds ratios for reporting different levels of problems and the most common health states in the population will be estimated. For study two, t-tests and ANOVA will be used to explore differences between groups in HRQoL and for each dimension of EQ-5D.

Ethics&dissemination

Ethics approval was given by the National Bioethics Committee of Medicines and Medical Devices Romania and Newcastle University's Research Ethics Committee.

Results will be published in peer-reviewed journals, presented at scientific conferences and on the project’s website. The EQ-5D-5L anonymised datasets will be deposited in a centralised repository. Two public workshops with local authorities, physicians and patients’ associations will be held.

Keywords: Health Economics, Health policy, Public Health, Statistics & Research Methods, Quality in health care

For peer review only - http://bmjopen.bmj.com/site/about/guidelines.xhtml

Article Summary

Strengths and limitations of this study

- First study to determine the value sets and population norms for both EQ-5D-3L and 5L for Romania.
- First study to determine HRQoL (as measured with EQ-5D-5L) of Roma population from Romania
- The representative samples include only persons who reside in the country at the time of the data collection. Romania has one of the highest migration rates in Europe: 17% of the total population of Romania (3.3 million of 19.6 million) are working abroad, many of them of Roma ethnicity.

For peer review only

For peer review only - http://bmjopen.bmj.com/site/about/guidelines.xhtml

Introduction

Health-Related Quality of Life (HRQL) can be described as a patient's emotional view on the effect illness and its associated treatment(s) can have on his/her everyday life, physical, mental, social functioning and prosperity [1].

In the last 20 years, the importance of HRQoL in health research has increased with many health care authorities recommending the use of indicators, such as quality-adjusted life years (QALYs), rather than relying on just indicators of mortality or morbidity alone [2]. QALYs are the mainstay of economic evaluations that help decision-makers determine how resources can be used to give the greatest benefits [3]. QALYs combine information on the length of life with information on quality of life, expressed as utilities. Several tools are available to measure individuals' health status and to attach utility values to these health states, such as the EQ-5D [4 5], Health Utilities Index (HUI) [6 7], Short Form 6-dimension (SF-6D)[8] and so on.

The EQ-5D is one of the most commonly used questionnaires to elicit health states utilities, having both a youth [9] and adult version. For general populations of adults two versions of the questionnaire have been developed, 3L and 5L, with the latter offering a more detailed description of health status [5]. In order to use the EQ-5D to estimate QALYs, a value set to reflect people's preferences for different health states is needed. At present, no value sets have ever been developed in Romania for either the 3L or 5L versions of the questionnaire. Researchers and decision makers have always used the value set from another jurisdiction, most commonly the value sets in the UK [10-13]. Using value sets from other countries might introduce bias as people's preferences can be culturally sensitive, differing from one country to another [14]. Therefore, local reimbursement and health technology assessment (HTA) decisions should be based on data applicable to the local context to avoid incorrect and inefficient decisions about the allocation of resources[15].

Besides their use in economic evaluations, tools such as the EQ-5D can be used to identify health problems in the community. The quality of life of patients or vulnerable groups can be compared with that of an average person from the population of interest with the help of population norms. This can help identify health inequalities between different groups. This in turn may help identify where health care and public health interventions are needed to reduce inequalities. This is of particular relevance to Romania where health inequalities have increased sharply in the past few years between regions and groups [16]. One particularly vulnerable group is represented by the Roma communities whose health status is consistently reported as being poorer than that of the general population [17]. Up to this date, the HRQoL of either the general population or Roma communities has never been determined with the EQ-5D questionnaire and no population norms exist in Romania for either of the two versions of the questionnaire.

For peer review only - http://bmjopen.bmj.com/site/about/guidelines.xhtml

The aim of this study is to determine the value sets and population norms for the EQ-5D questionnaires and to compare HRQoL (measured with EQ-5D-5L) between the general population and Roma communities.

Methods and analysis

I. Study design

A cross-sectional study design consisting of face-to-face interviews conducted in two national representative samples (general population and Roma communities) recruited from all regions of Romania will be conducted.

II. Aim and objectives

Our survey aims to provide HRQoL data to support HTA and reimbursement decisions in Romania and to support the development of interventions that are more responsive to the needs of local communities in Romania. Our specific objectives are:

1. To develop a value set for EQ-5D-3L and 5L based on societal preferences in Romania (Study one, an Omibus survey)
2. To determine the population norms of the Romanian version of EQ-5D-3L and 5L (Study one, an Omibus survey)
3. To describe differences in HRQoL between Roma communities and the majority of the population according to sociodemographic characteristics, socioeconomic status and ethnicity (Study two)

III. Study population

A. Target population

Study one

Non-institutionalized adults older than 18 who reside in the country at the time of the data collection.

Study two

Non-institutionalized self-identified Roma adults older than 18 who reside in the country at the time of the data collection.

B. Sampling frame

Study one & Study two

The sampling frame consists of all polling stations in Romania (18,626 polling stations for 3,181 settlements) that are publicly available on the website of the Romanian Permanent Electoral Authority.

C. Sample design

For peer review only - http://bmjopen.bmj.com/site/about/guidelines.xhtml

Study one

The sample was selected using a three-stage probability sampling procedure stratified by region and settlement size. Romania's territory was divided into strata using two criteria built according to the recommendations of the National Institute of Statistics:

1. Regional divisions (development regions):
   a. North-East: Bacău, Botoşani, Iaşi, Neamţ, Suceava, Vaslui
   b. South-East: Brăila, Buzău, Constanţa, Galaţi, Tulcea, Vrancea
   c. South: Argeş, Călăraşi, Dâmboviţa, Giurgiu, Ialomiţa, Prahova, Teleorman
   d. South-West: Dolj, Gorj, Mehedinţi, Olt, Vâlcea
   e. West: Arad, Caraş-Severin, Hunedoara, Timiş
   f. North-West: Bihor, Bistriţa-Năsăud, Cluj, Maramureş, Satu Mare, Sălaj
   g. Centre: Alba, Braşov, Covasna, Harghita, Mureş, Sibiu
   h. Bucharest: Ilfov, Bucharest
2. Size of settlement:
   a. Bucharest (more than 1 million inhabitants)
   b. Cities with more than 160.000 inhabitants, but up to 1 million inhabitants (≤
   c. Cities with 50.000 – 160.000 inhabitants
   d. Cities with less than 50.000 inhabitants
   e. Rural settlements

The strata were obtained by crossing the categories of the two criteria described above: a total of 19 strata was obtained (see Table 1). The first three categories of the "size of settlement" criteria were not crossed with the criteria "regional divisions" given that the number of settlements in the resulting strata would have been too small.

……………………….. ……… Insert table 1 about here.…………………………..

Table 1 – Number of sampled primary units by development region and settlement size

Within each resulting stratum, settlements were selected using a simple random sampling procedure without replacement. A total of 32 settlements was selected (see Figure 1): one settlement from the first bracket of the second criteria, eight from the second and third brackets of the second criteria, eight from the fourth bracket of the second criteria (one city with less than 50,000 inhabitants from each development region) and 15 from the last bracket (two rural settlements for the first seven development regions and one rural settlement for Bucharest). The sample size for each strata was determined based on disproportionate allocation to strata to ensure that at least 30 respondents would be selected for each settlement.

……………………….. ……… Insert figure 1 about here.…………………………..

For peer review only - http://bmjopen.bmj.com/site/about/guidelines.xhtml

Figure 1 – Geographical distribution of selected settlements according to development region and size

The second stage consists of selecting the polling stations that will be used as a starting point for the "random route" selection of participants. Polling stations were selected using a simple random sampling procedure without replacement for all settlements selected in the previous stage. The number of polling stations was determined so that a minimum of 20 people could be assigned to each polling station selected from a city and a minimum of 15 people to each polling station selected from a rural settlement. Hence, three polling stations were selected for cities with more than 50,000 inhabitants and two polling stations for cities with less than 50,000 inhabitants and rural settlements. Given that rural settlements can consist of one or more villages, the two polling stations were selected, if needed, from two different villages. In case of Bucharest, two polling sections were selected for each of the six sectors of the city. In total 82 polling stations were selected.

The third stage consists of selecting households and individuals from each settlement and "voting district". This will be done using a random route sampling method [18]. Interviewers will perform a random walk per "voting district". The interviewer will begin his/her route on the street whose name comes first in alphabetical order from the list of streets that make up the voting district. Once the street has been identified, he/she will start with the household located at the lowest household number on the street. To select the next household, the interviewer will walk on the same side of the street and select the 3rd household in ascending order.

In each household, the respondent will be selected based on "birthday rule": the adult whose birthday comes next from the interviewer's visit will be interviewed. In case of household non-response, the interviewer will visit the household two more times on different days and at different hours. If no contact is established after three calls, then the household is replaced with a new one as per the procedure described above (third household selected).

Study two

In order to increase the comparability of the two samples (general population and Roma communities) study two used the same sampling design as study one. Roma participants were selected from the same settlements selected for study one. If, based on 2011 census data, no Roma communities were reported to be living in the selected settlements, these were replaced with other settlements from the same strata with higher Roma representation. Disproportionate allocation to strata was used to select the number of respondents from each strata.

Households and individuals from each settlement will be selected either based the random route technique ("birthday rule") in those settlements that match the ones selected for study one and on a snowball technique for the rest. In case of non-response, the interviewer will make two more attempts on different days and at different hours. If no contact is established after three calls, then the household is replaced with a new one.

For peer review only - http://bmjopen.bmj.com/site/about/guidelines.xhtml

D. *Sample size calculation*

Study one

The minimum sample size needed to develop a value set for EQ-5D-3L is 300 people [19] and for EQ-5D-5L is 1,000 people [20]. Normative data for any outcome instrument needs to be obtained from a sample that is an accurate representation of the general population [21]. The sample size needed to select a national representative sample of non-institutionalised Romanian adults aged 18 and older was estimated to be 1,613 people, with a maximum error of ±3% at a confidence level of 95%. Due to the complex nature of the survey, the sample size required for a simple random sample (SRS) was adjusted by the design effect (deff) [22] and by a correction factor to account for the fact that only one person per household was selected (deff(p)). Given that neither the EQ-5D-3L or 5L has been used before to measure HRQoL in Romania, self-reported health, whereby respondents are asked to classify their current health status, was used as a proxy for HRQoL.

$$N = \frac{t^2 * p\,(1-p)}{\Delta^2} * deff * deff(p) = 896 * 1.5 * 1.2 = 1{,}613$$

t=1.96, given a 95% confidence interval; Δ = 0.03;

p= 0.7 (70% of Romanians considered having good health status according to the 2015 National Survey on Quality of Life) [23];

deff=1.5 [24]; deff (p)=1.2

The expected non-response rate was set at 10% leading to a final sample size of 1,794 people.

Study two

Sample size calculations for study two were estimated using the same formula described above leading to a sample size of 606 people, with a maximum error of ±5% at a confidence level of 95%. The sample size was similarly inflated to cover a 10% non-response rate leading to a target sample size of 673 people.

IV. *Pilot study*

Study one

The study's brochure, informed consent forms and the software needed to collect the data was pretested with approximately 10 volunteers for wording, clarity, order of questions and potential technical problems. Additionally, the local team of investigators performed approximately 30 interviews in Bucharest to gain experience with performing valuation task interviews.

For peer review only - http://bmjopen.bmj.com/site/about/guidelines.xhtml

All interviewers attended a two days and half face-to-face training in October 2018. Prior to the training, interviewers were asked to read the interviewer's guideline and to familiarize themselves with the data collection tools prior to the face-to-face training. At the end of the training, all interviewers performed approximately five interviews in Bucharest with a convenience sample of respondents selected from hospitals and schools. The local team of investigators assessed the quality of these pilot interviews and provide personalized feedback to each interviewer.

Study two

The study's brochure and informed consent forms were pretested with a group of five volunteers from Dâmbovița county and four health mediators. The survey company also pretested the contents of the survey for wording, order of questions and timings with approximately 10 volunteers from Cluj, a city in Romania. In collaboration with the local team of investigators, the survey company will provide an online training to all participating interviewers.

V. Data collection

Study one

Lay people will perform face-to-face interviews in respondents' homes using laptops connected to a secure online survey site (EQ-VT software version 2.1). Interviewers were selected from members of the national network of hospital assessors managed by the National Authority of Quality Management in Health from Romania and from members of patients' associations. In order to ease respondent recruitment, information about the study will be made available to the general public via social media and, where possible, more specific information will be given to the selected settlements via the Facebook page of the respective city hall (if available) or by contacting key community members.

A. Study questionnaire

The survey will consist of the following five block sections:

1. The EQ-5D-5L questionnaire and background questions on gender, age and experience of illness. EQ-5D-5L, one of the most widely used generic questionnaires to measure HRQoL [25], is a self-reported questionnaire that asks the respondent to assess his/her current health status on five dimensions (mobility, self-care, usual activities, pain/discomfort and anxiety/depression) and includes a visual analogue scale (EQ-VAS).
2. composite Time-Trade Off (cTTO) valuations tasks (EQ-5D-3L and 5L). In this section respondents will be asked to imagine living in an impaired health state for a defined period of time and indicate the years of remaining lifetime they will be willing to give up on to avoid living in the impaired health state. Respondents will complete two examples

For peer review only - http://bmjopen.bmj.com/site/about/guidelines.xhtml

and three practice health states to get used to the task. Then they will assess ten EQ-5D-5L health states and three EQ-5D-3L health states each followed by a feedback module.

3. Discrete choice valuation (DCE) tasks (only for EQ-5D-5L). Respondents will be asked to state their preference between two hypothetical health states. They will value seven pairs of EQ-5D-5L health states followed by debriefing questions on the valuation tasks.
4. The EQ-5D-3L questionnaire. Respondents will be asked to fill in the EQ-5D-3L questionnaire. EQ-5D-3L measures health status using the same five dimensions as the 5L version, the only difference being that each dimension has only three levels (no problems", "some problems", and "extreme problems).
5. Country specific questionnaire. The survey will end with a series of sociodemographic questions on residence type, ethnicity, caregiver and parenting status, health literacy (one question that explores the respondent's level of confidence when interacting with health care professionals), preference over length or quality of life, marital status, education level, religion (affiliation, general religiosity, participation in religious services, praying), employment status and income.

B. Data quality control

The data quality control process will follow the recommendations of the most up-to-date version of the EQ-VT protocol [26 27]. An online Quality Control (QC) tool developed by the EuroQoL foundation will be used to evaluate interviewers' performance. This tool determines protocol compliance, interviewers' effects and mean values by health state severity [28] and is used by the local team of investigators to provide personalized feedback via phone calls to all interviewers. Data quality control checks will be run after each interviewer will have performed a round of 10 interviews. Poor performing interviewers will be re-trained and removed from the team if no improvement is seen after re-training. Checks on the "random-walk" implementation will also be performed. The local team of investigators will contact by phone a random selection of respondents (10%) to verify that interviews did take place.

Study two

Interviews will be conducted by professional staff from a survey company with experience in working with the Roma community. They will be face-to-face, pen and pencil interviews. Interviewers' access to communities will be facilitated by contacting leaders of the respective communities such as health mediators, social worker assistants or school mediators. All interviews will be conducted with care and sensitivity and respect for all participants' level of literacy. The study will be presented in simple words and if the participant can't write, the interviewer will sign the informed consent on his/her behalf if he/she desires to take part in the study.

For peer review only - http://bmjopen.bmj.com/site/about/guidelines.xhtml

To ease recruitment, just like for study one, information about the study will be made available to the general public via social media (Facebook page, project webpage) and, if possible, more specific information will be made available to the city halls or health mediators of the selected settlements.

A. Study questionnaire

The survey will consist of the following two sections:

1. The EQ-5D-5L questionnaire
2. Sociodemographic questionnaire. This will include the same questions as the Country specific questionnaire used in study one with the addition of two more questions: the availability of health mediators in the respective community and the ability of the respondent to read and write.

B. Data quality control

The data quality control process will be performed by the survey company in accordance with its internal procedures. Paper questionnaires will be validated on a rolling basis and incomplete questionnaires will undergo a "data recovery process" by re-contacting respondents and resuming unanswered items. Additionally, a random selection of 20% of respondents will be contacted by phone to verify that interviews did take place. If needed, field checks will also be performed.

VI. Analysis

Study one

The EQ-5D-3L value set will be developed using only cTTO data whereas the 5L value set will use both cTTO and DCE data. Different models, such as ordinary least squares (OLS), generalized least squares (GLS) and tobit, will be used to analyse cTTO data, whereas probit or logit models will be used to analyse the DCE data. A hybrid model that uses both cTTO and DCE data will also be developed for the EQ-5D-5L value set. The model with the best fit will be chosen following theoretical considerations and comparisons of models based on the monotonic structure of the decrements, the standard errors, the ranking of the dimensions as well as the resulting predictions.

Population norms for both questionnaires will determined using the respective value sets. The distribution of people and the odds ratios for reporting different levels of problems for all dimensions of the two EQ-5D questionnaires will be calculated and stratified by sociodemographic variables. The frequency of the most common health states in the general population in Romania will also be determined.

The effects of different variables, such as health literacy or religion, on health valuations will also be explored. This will be done using simple and multiple linear regression models that will have as dependent variable the TTO scores. Other explanatory variables considered for these models will be age, sex, place of residence (urban/rural), and educational level. All models will be adjusted for

For peer review only - http://bmjopen.bmj.com/site/about/guidelines.xhtml

severity of health states with random effects to account for multiple valuations by the same respondent.

Study two

Potential differences in HRQoL according to sociodemographic characteristics and ethnicity will be explored in a subset of age and gender-matched sample of Roma population (study two) and general population (study one). T-tests and ANOVA analysis will be used to reflect differences across groups in HRQoL and for each dimension of the EQ-5D-5L questionnaire. Multiple regression analysis will be used to study how the EQ-5D-5L visual analogue scale varies with sociodemographic characteristics, educational level, occupation, income, self-rated health and chronic diseases in the two populations.

For both study one and two, the survey sample distributions will be adjusted by applying weights based on 2011 census data to the age, sex and regional distribution of the two samples.

Discussion

Our study will estimate for the first time in Romania value sets and population norms for the EQ-5D questionnaires. This will constitute a stepping-stone to further development of HTA in Romania as it will potentially lead to more transparent and consistent decision-making in healthcare and to a more efficient use of relatively scarce local resources. It will also provide for the first time a measure of the health status of the general population in Romania and that of Roma communities. This could feed into better public health interventions and policies for either vulnerable or patient groups. Finally, if sample size targets are met, this could become the largest study conducted in Romania on HRQoL.

However, our study has a certain number of limitations. First of all, our respondents will be selected using “the birthday rule”. “The birthday rule” is considered to be a quasi-probability procedure of selecting respondents [29], but it is the easiest and least time-consuming in terms of interviewers’ training and administration. It has also been implemented with good results in other representative surveys in Romania and elsewhere [30].

The survey weights that we will be using to correct the representativeness of the study’s samples will be estimated based on the most recent census data available, more exactly the 2011 census data. Since 2011, migration rates have been increasing in Romania with the country currently having the highest growth in the size of its diaspora population after Syria [31]. Unfortunately, the 2011 census data has not yet been updated with the most recent data on the exact number of Romanian emigrants [32 33], so our weights might not accurately correct the representativeness of the two samples. Also, due to the current troubled political context in Romania, our interviewers might be faced in the field with a lower propensity of local residents to participate in surveys. This could lead to lower than

For peer review only - http://bmjopen.bmj.com/site/about/guidelines.xhtml

predicted response rates for certain areas, such as urban areas, potentially affecting the quality of the data through non-response bias.

Finally, our survey on Roma communities will consist of self-identified Roma adults. Several studies in Romania have shown that self-identification and hetero-identification generally do not overlap [34 35]. Moreover, self-identified Roma tend to be more impoverished and with a higher residential segregation than hetero-identified Roma [34]. Hence, our comparison results might overestimate the differences between the two groups.

Ethics and dissemination

This study will be conducted in agreement with ethical principles set out in the Declaration of Helsinki and Regulation (EU) 679/2016 on the protection of natural persons with regard to the processing of personal data and the free movement of such data. Ethical approval was sought from the Bioethics Committee of Medicines and Medical Devices, Romania and from the Faculty of Medical Sciences Research Ethics Committee, part of Newcastle University's Research Ethics Committee, United Kingdom. As the project involves working with a vulnerable group, the project is of high risk in terms of ethics. Hence, all interviews will be conducted with care and sensitivity and with respect for participants' ethnicity, religion, language, sexual orientation or literacy level. All participants will be given enough time to read or be read the study's information brochure and to ask questions and discuss concerns regarding potential participation in the study. All the study's materials will have been pretested with volunteers and/or Roma mediators with the aim to try to ensure that information is simple and that words are easy to understand. Participants will be presented the study's brochure, sign the informed consent forms, and be interviewed, all within one visit.

The dissemination plan for this project includes deliverables for the scientific community (scientific articles and conference presentations) and for the general public, such as press releases, brief communications on the project's website, Twitter and Facebook pages. Also, two public workshops with local authorities, physicians and patients associations will be organised to disseminate the results of the study to a broader audience. Finally, the anonymised datasets with EQ-5D-5L measures will be deposited in a centralised repository, such as Zenodo.

Bibliography

1. Agency EM. Reflection Paper on the Regulatory Guidance for the Use of Health- Related Quality of Life (Hrql) Measures in the Evaluation of Medicinal Products: European Medicines Agency, 2005.
2. Romero M, Vivas-Consuelo D, Alvis-Guzman N. Is Health Related Quality of Life (HRQoL) a valid indicator for health systems evaluation? *Springerplus* 2013;2:664. doi: 10.1186/2193-1801-2-664 [published Online First: 2013/12/20]

For peer review only - http://bmjopen.bmj.com/site/about/guidelines.xhtml

3. Cunningham SJ. Economic evaluation of healthcare--is it important to us? *Br Dent J* 2000;188(5):250-4. [published Online First: 2000/04/12]
4. Herdman M, Gudex C, Lloyd A, et al. Development and preliminary testing of the new five-level version of EQ-5D (EQ-5D-5L). *Qual Life Res* 2011;20(10):1727-36. doi: 10.1007/s11136-011-9903-x [published Online First: 2011/04/12]
5. EuroQol G. EuroQol--a new facility for the measurement of health-related quality of life. *Health Policy* 1990;16(3):199-208. [published Online First: 1990/11/05]
6. Boyle MH, Torrance GW. Developing multiattribute health indexes. *Med Care* 1984;22(11):1045-57. [published Online First: 1984/11/01]
7. Feeny D, Furlong W, Boyle M, et al. Multi-attribute health status classification systems. Health Utilities Index. *Pharmacoeconomics* 1995;7(6):490-502. doi: 10.2165/00019053-199507060-00004 [published Online First: 1995/05/08]
8. Brazier J, Roberts J, Deverill M. The estimation of a preference-based measure of health from the SF-36. *J Health Econ* 2002;21(2):271-92. [published Online First: 2002/04/10]
9. Wille N, Badia X, Bonsel G, et al. Development of the EQ-5D-Y: a child-friendly version of the EQ-5D. *Qual Life Res* 2010;19(6):875-86. doi: 10.1007/s11136-010-9648-y [published Online First: 2010/04/21]
10. Mogosan C, Stoica V, Mihai C, et al. Trends of rheumatoid arthritis monitorization in Romania. *J Med Life* 2010;3(3):330-7. [published Online First: 2010/10/16]
11. De Smedt D, Clays E, Doyle F, et al. Validity and reliability of three commonly used quality of life measures in a large European population of coronary heart disease patients. *Int J Cardiol* 2013;167(5):2294-9. doi: 10.1016/j.ijcard.2012.06.025 [published Online First: 2012/07/04]
12. De Smedt D, Clays E, Hofer S, et al. Validity and reliability of the HeartQoL questionnaire in a large sample of stable coronary patients: The EUROASPIRE IV Study of the European Society of Cardiology. *Eur J Prev Cardiol* 2016;23(7):714-21. doi: 10.1177/2047487315604837 [published Online First: 2015/09/12]
13. Paveliu M, Lorenzovici L, Tudose C. Oral anticoagulant treatment with antivitamin K in romania. A cost-effectiveness analysis of using INR home testing devices. *Farmacia* 2018;66(2):358 - 64.
14. Knies S, Evers SM, Candel MJ, et al. Utilities of the EQ-5D: transferable or not? *Pharmacoeconomics* 2009;27(9):767-79. doi: 10.2165/11314120-000000000-00000 [published Online First: 2009/09/18]
15. Eurostat. Healthcare expenditure statistics, 2017.
16. Precupetu I, Precupetu M. GINI Country Report: Growing Inequalities and their Impacts in Romania. GINI Country Reports: AIAS, Amsterdam Institute for Advanced Labour Studies, 2013.
17. Cook B, Wayne GF, Valentine A, et al. Revisiting the evidence on health and health care disparities among the Roma: a systematic review 2003-2012. *Int J Public Health* 2013;58(6):885-911. doi: 10.1007/s00038-013-0518-6 [published Online First: 2013/10/08]
18. Hoffmeyer-Zlotnik JH, Krebs D. Different methods of survey sampling in Germany. In: Ferligoj A, Kramberger A, eds. Developments in Data Analysis. Ljubljana: Metodoloski zvezki 1996.
19. Lamers LM, McDonnell J, Stalmeier PF, et al. The Dutch tariff: results and arguments for an effective design for national EQ-5D valuation studies. *Health economics* 2006;15(10):1121-32. doi: 10.1002/hec.1124
20. Oppe M, van Hout B. The “power” of eliciting EQ-5D-5L values. *EuroQoL Working Paper Series* 2017
21. Nolte MT, Shauver MJ, Chung KC. Analysis of four recruitment methods for obtaining normative data through a Web-based questionnaire: a pilot study. *Hand (N Y)* 2015;10(3):529-34. doi: 10.1007/s11552-014-9730-y
22. Laaksonen S. Sampling and estimation of multi-national surveys with examples from the European Social Survey, 2006.
23. National Institute of Statistics. Survey on quality of life. Romania. 2015
24. Rizzo C, Fabiani M, Amlot R, et al. Survey on the Likely Behavioural Changes of the General Public in Four European Countries During the 2009/2010 Pandemic. In: Manfredi P,

For peer review only - http://bmjopen.bmj.com/site/about/guidelines.xhtml

D'Onofrio A, eds. Modeling the Interplay Between Human Behavior and the Spread of Infectious Diseases: Springer, New York, NY 2012.
25. Gusi N, Olivares PR, Rajendram R. The EQ-5D Health-Related Quality of Life Questionnaire. In: Preedy VR, Watson RR, eds. Handbook of Disease Burdens and Quality of Life Measures. New York, NY: Springer 2010.
26. Stolk EA, Ludwig K, Rand-Hendriksen K, et al. Overview, update and lessons learned from the international EQ-5D-5L valuation work: version 2 of the EQ-5D-5L valuation protocol *Value Health (submitted)*
27. Oppe M, Devlin NJ, van Hout B, et al. A program of methodological research to arrive at the new international EQ-5D-5L valuation protocol. *Value in health : the journal of the International Society for Pharmacoeconomics and Outcomes Research* 2014;17(4):445-53. doi: 10.1016/j.jval.2014.04.002
28. Ramos-Goni JM, Oppe M, Slaap B, et al. Quality Control Process for EQ-5D-5L Valuation Studies. *Value Health* 2017;20(3):466-73. doi: 10.1016/j.jval.2016.10.012
29. Salmon CT, Nichols JS. The Next-Birthday Method of Respondent Selection. *Public Opin Quart* 1983;47(2):270-76. doi: Doi 10.1086/268785
30. Eurofund. 3rd European Quality of Life Survey: sampling report EU27 and non-EU countries: Eurofund, 2012.
31. United Nations DoEaSA, Population Division. International Migration Report 2015: Highlights (ST/ESA/SER.A/375), 2016.
32. Pisica S, Caragea N. Statistical overview of the international labor migration in Romania: Working papers 08, Ecological University of Bucharest, 2013.
33. Prada E, Ciupureanu C-A, Otoiu A. A Regional Analysis of Romanian Migration Determinants2015.
34. Ladányi J, Szelenyi I. The social construction of Roma ethnicity in Bulgaria, Romania and Hungary during market transition2001.
35. Cace S, Toader R, Vizireanu A. Romii din România. De la țap ispășitor la motor de dezvoltare. In: Gelu D, Ana I, eds.: Agentia Impreuna, 2013.

Acknowledgements

The authors would like to thank Kristina Ludwig and Elly Stolk from the EuroQoL Research foundation for their comments on the protocol of the general population study (Study one) and support offered during the setup of study one. We would also like to thank Jan Deckers for his helpful comments and support in preparing the ethical submission for this project.

Authors’ contributions

The study was conceived by IGNA, EO, YO, LV, EB, and SP. EO wrote the first draft of the manuscript. All authors provided their intellectual input and approved the final manuscript.

Funding

These surveys are conducted by two teams of researchers that applied independently for funding. Given that several support activities are common across surveys, the two teams decided to offer each other methodological and scientific support. Hence, these surveys have several funding sources. The estimation of EQ-5D-5L value set, population norms and the comparison with Roma communities received funding from the European Union’s Horizon 2020 research and innovation programme under the Marie Skłodowska-Curie grant agreement No 748612. It has also received

For peer review only - http://bmjopen.bmj.com/site/about/guidelines.xhtml

funding from the EuroQol Research Foundation. The estimation of EQ-5D-3L value set and population norms received partial funding as part of a project run through the Romanian Operational Programme “Administrative Capacity” (POCA 2014-2020).

Competing interests

None.

Patient consent

Not required.

Ethics approval

The study protocol was reviewed and approved by the Bioethics Committee of Medicines and Medical Devices, Romania and by the Faculty of Medical Sciences Research Ethics Committee, part of Newcastle University's Research Ethics Committee, United Kingdom.

Data sharing statement

Not required at protocol stage.

For peer review only

Strata No.	Strata label	Number of settlements (primary units) in the population	Number of settlements (primary units) in the sample
1	Cities > 1 mil inh	1	1
2	160.000 inh. ≤ cities < 1 mil inh	11	4
3	50.000 inh. ≤ cities <160.000 inh.	29	4
4	South: cities < 50.000 inh.	43	1
5	South-East: cities < 50.000 inh	35	1
6	West: cities < 50.000 inh	37	1
7	Centre: cities < 50.000 inh	52	1
8	North-West: cities < 50.000 inh	37	1
9	North-East: cities < 50.000 inh	38	1
10	South-East: cities < 50.000 inh	29	1
11	Bucharest-Ilfov: cities < 50.000 inh	8	1
12	South-East: rural settlements	519	2
13	South: rural settlements	408	2
14	West: rural settlements	281	2
15	Centre: rural settlements	357	2
16	North-West: rural settlements	403	2
17	North-East: rural settlements	506	2
18	South-East: rural settlements	355	2
19	Bucharest-Ilfov: rural settlements	32	1
	**Total**	**3181**	**32**

Mil: million; inh: Inhabitants;

Table 1 - Number of sampled primary units by development region and settlement size

For peer review only - http://bmjopen.bmj.com/site/about/guidelines.xhtml

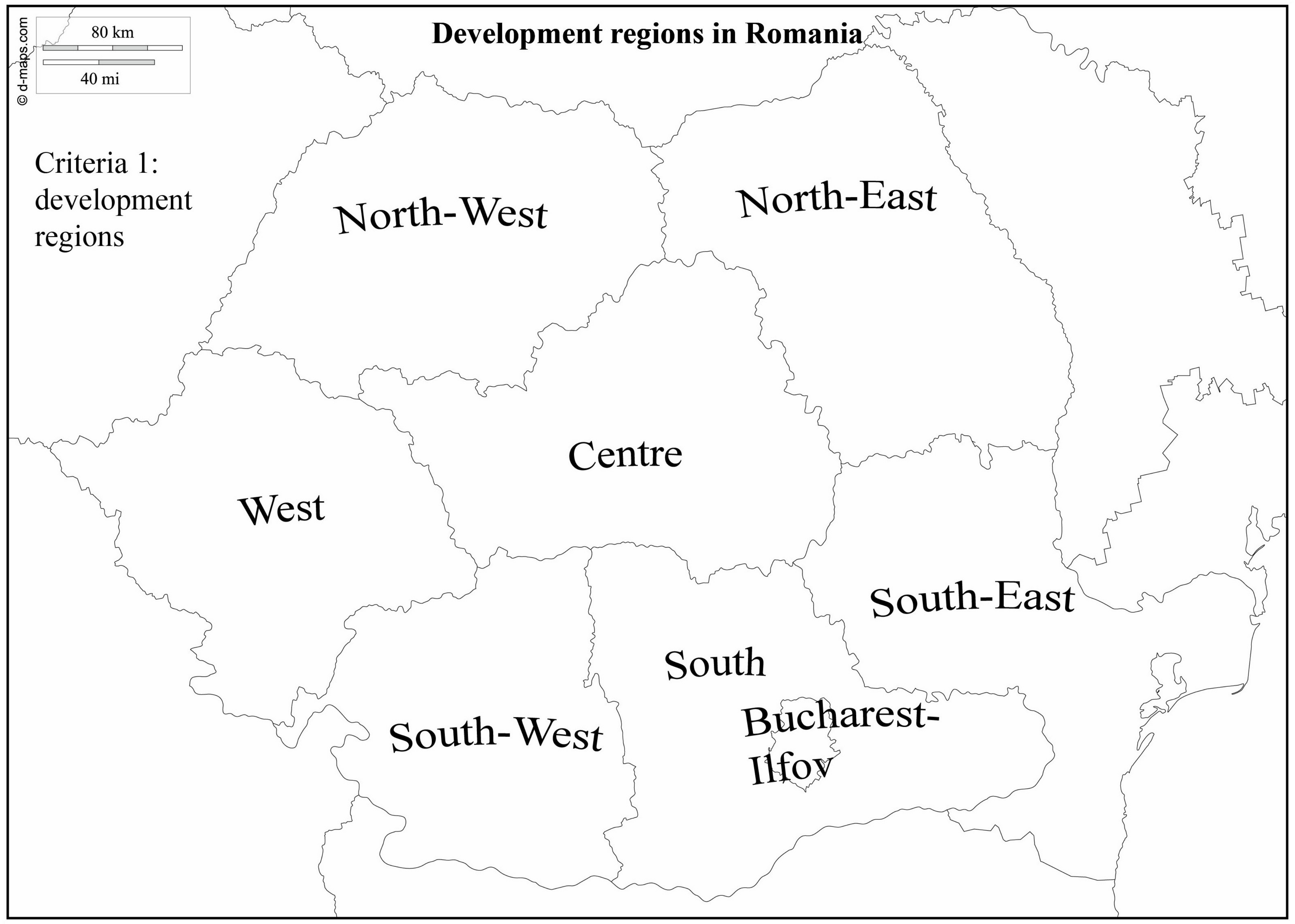

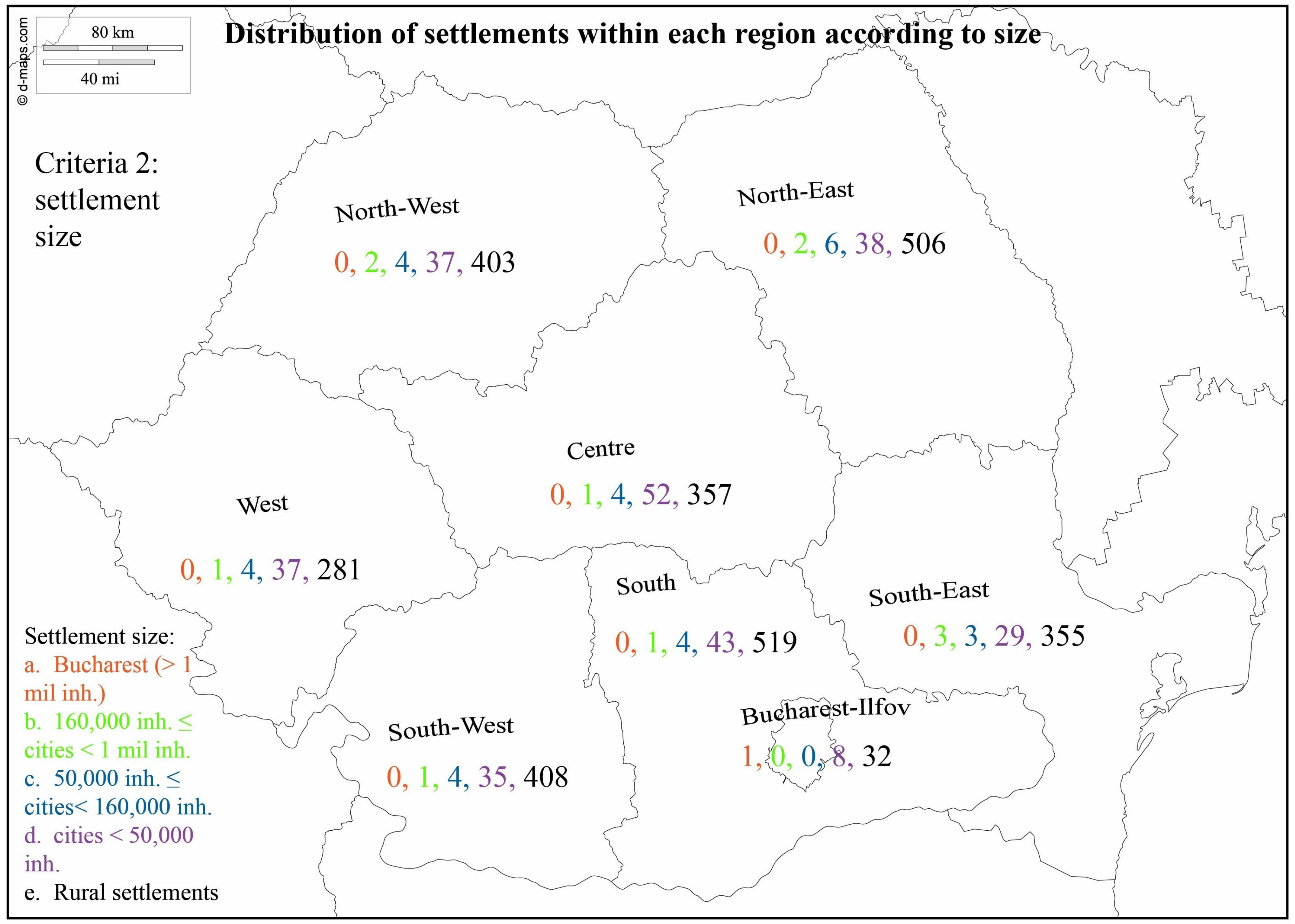

Criteria 1 + Criteria 2

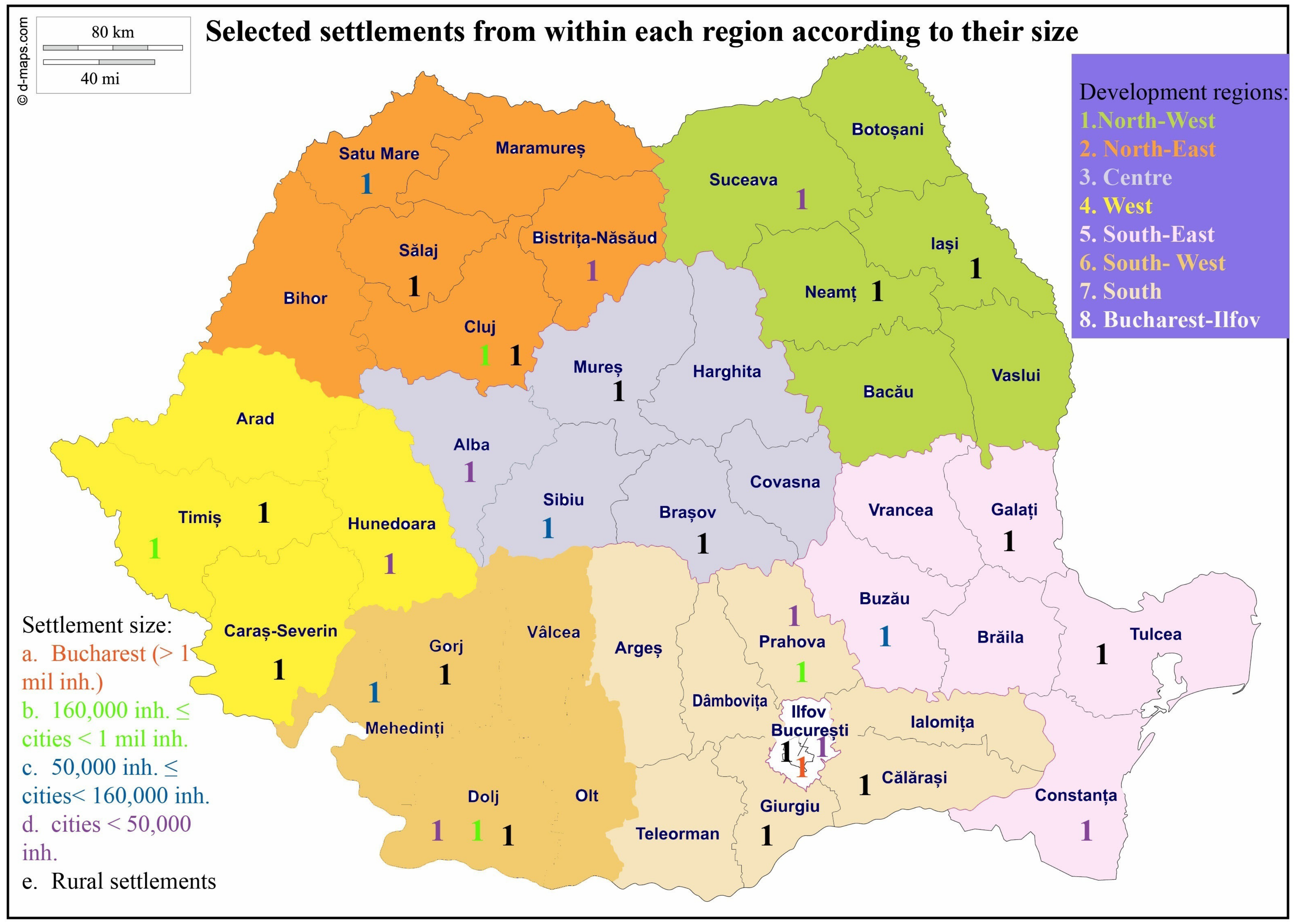

Original maps downloaded from https://d-maps.com/carte.php?num_car=5805&lang=en and https://d-maps.com/carte.php?num_car=25497&lang=en. Edited by EO.

BMJ Open

Measuring health-related quality of life in the general population and Roma communities in Romania: study protocol for two cross-sectional studies

Journal:	*BMJ Open*
Manuscript ID	bmjopen-2019-029067.R1
Article Type:	Protocol
Date Submitted by the Author:	27-May-2019
Complete List of Authors:	Olariu, Elena; Newcastle University, Institute of Health and Society<br>Paveliu, Sorin; Universitatea Titu Maiorescu Facultatea de Medicina, Department of Pharmacology and Pharmaeconomics<br>Baican, Eugen; Universitatea Babes-Bolyai, Department of Social Work<br>Oluboyede , Yemi; Newcastle University, Health Economics Group, Institute of Health and Society<br>Vale, Luke; Newcastle University, Health Economics Group, Institute of Health and Society<br>NICULESCU-ARON, ILEANA GABRIELA ; Academia de Studii Economice din Bucuresti, Department of Statistics and Econometrics, Faculty of Economic Cybernetics, Statistics and Informatics
**Primary Subject Heading**:	Health economics
Secondary Subject Heading:	Health policy
Keywords:	HEALTH ECONOMICS, Health policy < HEALTH SERVICES ADMINISTRATION & MANAGEMENT, Quality in health care < HEALTH SERVICES ADMINISTRATION & MANAGEMENT, PUBLIC HEALTH, STATISTICS & RESEARCH METHODS

SCHOLARONE™
Manuscripts

Measuring health-related quality of life in the general population and Roma communities in Romania: study protocol for two cross-sectional studies

Elena Olariu[1] , Sorin Paveliu[2]*, Eugen Baican[3], Yemi Oluboyede[1], Luke Vale[1], Ileana Gabriela Niculescu-Aron[4]

[1] Health Economics Group, Institute of Health and Society, Newcastle University, Newcastle upon Tyne, United Kingdom

[2] Department of Pharmacology and Pharmaeconomics, Faculty of General Medicine, Titu Maiorescu University, Bucharest, Romania

[3] Department of Social Work, Babes-Bolyai University, Cluj-Napoca, Romania

[4] Department of Statistics and Econometrics, Faculty of Economic Cybernetics, Statistics and Informatics, Bucharest University of Economic Studies, Bucharest, Romania

* Corresponding author

Address for correspondence: Sorin Paveliu MD, PhD, Faculty of General Medicine, Titu Maiorescu University, 67A Gheorghe Petrascu Str., district 3, Bucharest, code 031593, Romania. Email: sorinpaveliu@yahoo.com. Telephone: 0040726.200.627.

Word count: 3,968 words

For peer review only

For peer review only - http://bmjopen.bmj.com/site/about/guidelines.xhtml

Abstract

Introduction

The importance of health-related quality of life (HRQoL) is increasing and many healthcare authorities recommend the use of measures that account for both mortality and morbidity. This study will determine, for the first time in Romania, value sets for EQ-5D-3L and EQ-5D-5L questionnaires and their population norms (study one). It will also compare the HRQoL (measured with EQ-5D-5L) of Roma communities in Romania with that of the general population (study two).

Methods & analysis

Cross-sectional studies of face-to-face interviews conducted in representative samples of the Romanian general population and Romanian Roma communities. 1,614 non-institutionalized adults older than 18 will be interviewed using a computer-assisted interview for study one. Participants will complete EQ-5D-3L and 5L, 13 composite time-trade-off tasks (cTTO), seven discrete choice experiment questions (DCE) and sociodemographic questions. For study two, 606 non-institutionalized self-identified Roma people older than 18 will be interviewed using a pencil-and-paper interview. Participants will complete EQ-5D-5L and the same sociodemographic questions as for study one.

The 3L value set will be estimated using econometric models and the cTTO data. cTTO and DCE data will be used for the 5L value set. Population norms will be reported by age and gender. The odds ratios for reporting different levels of problems and the most common health states in the population will be estimated. For study two, t-tests and ANOVA will be used to explore differences between groups in HRQoL and for each EQ-5D dimension.

Ethics & dissemination

Ethics approval was given by the National Bioethics Committee of Medicines and Medical Devices Romania and Newcastle University's Research Ethics Committee.

Results will be published in peer-reviewed journals, presented at scientific conferences and on the project's website. The EQ-5D-5L anonymised datasets will be deposited in a centralised repository. Two public workshops with local authorities, physicians and patients' associations will be held.

Keywords: Health Economics, Health policy, Public Health, Statistics & Research Methods, Quality in healthcare

For peer review only

For peer review only - http://bmjopen.bmj.com/site/about/guidelines.xhtml

Article Summary

Strengths and limitations of this study

- First study to determine the value sets and population norms for both EQ-5D-3L and 5L for Romania.
- First study to determine HRQoL (as measured with EQ-5D-5L) of Roma population from Romania
- The representative samples include only persons who reside in the country at the time of the data collection. Romania has one of the highest migration rates in Europe: 17% of the total population of Romania (3.3 million of 19.6 million) are working abroad, many of them of Roma ethnicity.

For peer review only

For peer review only - http://bmjopen.bmj.com/site/about/guidelines.xhtml

Introduction

Health-Related Quality of Life (HRQoL) is a multidimensional concept that includes physical, psychological, functional, and social aspects [1] regarding a person's perception of the impact of health status on quality of life [2].

In the last 20 years, the importance of HRQoL in health research has increased with many healthcare authorities recommending the use of indicators, such as quality-adjusted life years (QALYs), rather than relying on just indicators of mortality or morbidity alone [3]**.** QALYs are the mainstay of economic evaluations that help decision-makers determine how resources can be used to give the greatest benefits [4]. QALYs combine information on the length of life with information on quality of life, expressed as utilities. Several tools are available to measure individuals' health status and to attach utility values to these health states, such as the EQ-5D [5 6], Health Utilities Index (HUI) [7 8], Short Form 6-dimension (SF-6D)[9] and so on.

The EQ-5D is one of the most commonly used questionnaires to elicit health states utilities, having both a youth [10] and adult version. For general populations of adults two versions of the questionnaire have been developed, 3L and 5L, with the latter offering a more detailed description of health status [6]. In order to use the EQ-5D to estimate QALYs, a value set to reflect people's preferences for different health states is needed. At present, no value sets have ever been developed in Romania for either the 3L or 5L versions of the questionnaire. Researchers and decision makers have always used the value set from another jurisdiction, most commonly the value sets in the UK [11-14]. Using value sets from other countries might introduce bias as people's preferences can be culturally sensitive, differing from one country to another [15]. Therefore, local reimbursement and health technology assessment (HTA) decisions should be based on data applicable to the local context to avoid incorrect and inefficient decisions about the allocation of resources[16].

Besides their use in economic evaluations, tools such as the EQ-5D can be used to identify health problems in the community. The quality of life of patients or vulnerable groups can be compared with that of an average person from the population of interest with the help of population norms. This can help identify health inequalities between different groups. This in turn may help identify where healthcare and public health interventions are needed to reduce inequalities. This is of particular relevance to Romania where health inequalities have increased sharply in the past few years between regions and groups [17]. One particularly vulnerable group is represented by the Roma communities whose health status is consistently reported as being poorer than that of the general population [18]. Up to this date, the HRQoL of either the general population or Roma communities has never been determined with the EQ-5D questionnaire and no population norms exist in Romania for either of the two versions of the questionnaire.

For peer review only - http://bmjopen.bmj.com/site/about/guidelines.xhtml

The aim of this study is to determine the value sets and population norms for the EQ-5D questionnaires and to compare HRQoL (measured with EQ-5D-5L) between the general population and Roma communities.

Methods and analysis

I. Study design

A cross-sectional study design consisting of face-to-face interviews conducted in two national representative samples (general population and Roma communities) recruited from all regions of Romania will be conducted from November 2018 to April 2019.

II. Aim and objectives

Our survey aims to provide HRQoL data to support HTA and reimbursement decisions in Romania and to support the development of interventions that are more responsive to the needs of local communities in Romania. Our specific objectives are:

1. To develop a value set for EQ-5D-3L and 5L based on societal preferences in Romania (Study one, an Omnibus survey)
2. To determine the population norms of the Romanian version of EQ-5D-3L and 5L (Study one, an Omnibus survey)
3. To describe differences in HRQoL between Roma communities and the majority of the population according to sociodemographic characteristics, socioeconomic status and ethnicity (Study two)

III. Study population

A. Target population

Study one

Non-institutionalized adults older than 18 who reside in the country at the time of the data collection.

Study two

Non-institutionalized self-identified Roma adults older than 18 who reside in the country at the time of the data collection.

B. Sampling frame

Study one & Study two

The sampling frame consists of all polling stations in Romania (18,626 polling stations for 3,181 settlements) that are publicly available on the website of the Romanian Permanent Electoral Authority.

C. Sample design

For peer review only - http://bmjopen.bmj.com/site/about/guidelines.xhtml

Study one

The sample was selected using a three-stage probability sampling procedure stratified by region and settlement size. Romania's territory was divided into strata using two criteria built according to the recommendations of the National Institute of Statistics:

1. Regional divisions (development regions):
    a. North-East: Bacău, Botoşani, Iaşi, Neamţ, Suceava, Vaslui
    b. South-East: Brăila, Buzău, Constanţa, Galaţi, Tulcea, Vrancea
    c. South: Argeş, Călăraşi, Dâmboviţa, Giurgiu, Ialomiţa, Prahova, Teleorman
    d. South-West: Dolj, Gorj, Mehedinţi, Olt, Vâlcea
    e. West: Arad, Caraş-Severin, Hunedoara, Timiş
    f. North-West: Bihor, Bistriţa-Năsăud, Cluj, Maramureş, Satu Mare, Sălaj
    g. Centre: Alba, Braşov, Covasna, Harghita, Mureş, Sibiu
    h. Bucharest: Ilfov, Bucharest
2. Size of settlement:
    a. Bucharest (more than 1 million inhabitants)
    b. Cities with more than 160.000 inhabitants, but up to 1 million inhabitants (≤
    c. Cities with 50.000 – 160.000 inhabitants
    d. Cities with less than 50.000 inhabitants
    e. Rural settlements

The strata were obtained by crossing the categories of the two criteria described above: a total of 19 strata was obtained (see Table 1). The first three categories of the "size of settlement" criteria were not crossed with the criteria "regional divisions" given that the number of settlements in the resulting strata would have been too small.

Strata No.	***Strata label***	***Number of settlements (primary units) in the population***	***Number of settlements (primary units) in the sample***
1	Cities > 1 mil inh	1	1
2	160.000 inh. ≤ cities < 1 mil inh	11	4
3	50.000 inh. ≤ cities <160.000 inh.	29	4
4	South: cities < 50.000 inh.	43	1
5	South-East: cities < 50.000 inh	35	1
6	West: cities < 50.000 inh	37	1
7	Centre: cities < 50.000 inh	52	1

For peer review only - http://bmjopen.bmj.com/site/about/guidelines.xhtml

Strata No.	Strata label	Number of settlements (primary units) in the population	Number of settlements (primary units) in the sample
8	North-West: cities < 50.000 inh	37	1
9	North-East: cities < 50.000 inh	38	1
10	South-East: cities < 50.000 inh	29	1
11	Bucharest-Ilfov: cities < 50.000 inh	8	1
12	South-East: rural settlements	519	2
13	South: rural settlements	408	2
14	West: rural settlements	281	2
15	Centre: rural settlements	357	2
16	North-West: rural settlements	403	2
17	North-East: rural settlements	506	2
18	South-East: rural settlements	355	2
19	Bucharest-Ilfov: rural settlements	32	1
	**Total**	**3181**	**32**

Mil: million; inh: Inhabitants

Table 1 – Number of sampled primary units by development region and settlement size

Within each resulting stratum, settlements were selected using a simple random sampling procedure without replacement. A total of 32 settlements was selected (see

Figure 1): one settlement from the first bracket of the second criteria, eight from the second and third brackets of the second criteria, eight from the fourth bracket of the second criteria (one city with less than 50,000 inhabitants from each development region) and 15 from the last bracket (two rural settlements for the first seven development regions and one rural settlement for Bucharest). The sample size for each strata was determined based on disproportionate allocation to strata to ensure that at least 30 respondents would be selected for each settlement.

……………………….. ……… Insert figure 1 about here.………………………..

Figure 1Geographical distribution of selected settlements according to development region and size

The second stage consists of selecting the polling stations that will be used as a starting point for the "random route" selection of participants. Polling stations were selected using a simple random sampling procedure without replacement for all settlements selected in the previous stage. The number of polling stations was determined so that a minimum of 20 people could be assigned to each polling station selected from a city and a minimum of 15 people to each polling station selected from a rural settlement. Hence, three polling stations were selected for cities with more than 50,000 inhabitants and two polling stations for cities with less than 50,000 inhabitants and rural settlements.

For peer review only - http://bmjopen.bmj.com/site/about/guidelines.xhtml

Given that rural settlements can consist of one or more villages, the two polling stations were selected, if needed, from two different villages. In case of Bucharest, two polling sections were selected for each of the six sectors of the city. In total 82 polling stations were selected.

The third stage consists of selecting households and individuals from each settlement and "voting district". This will be done using a random route sampling method [19]. Interviewers will perform a random walk per "voting district". The interviewer will begin his/her route on the street whose name comes first in alphabetical order from the list of streets that make up the voting district. Once the street has been identified, he/she will start with the household located at the lowest house number on the street. To select the next household, the interviewer will walk on the same side of the street and select the 3rd household in ascending order.

In each household, the respondent will be selected based on "birthday rule": the adult whose birthday comes next from the interviewer's visit will be interviewed. In case of household non-response, the interviewer will visit the household two more times on different days and at different hours. If no contact is established after three calls, then the household is replaced with a new one as per the procedure described above (third household selected).

Study two

In order to increase the comparability of the two samples (general population and Roma communities) study two used the same sampling design as study one. Roma participants were selected from the same settlements selected for study one. If, based on 2011 census data, no Roma communities were reported to be living in the selected settlements, these were replaced with other settlements from the same strata with higher Roma representation. Disproportionate allocation to strata was used to select the number of respondents from each stratum.

Households and individuals from each settlement will be selected either based on the random route technique ("birthday rule") in those settlements that match the ones selected for study one and on a snowball technique for the rest. In case of non-response, the interviewer will make two more attempts on different days and at different hours. If no contact is established after three calls, then the household is replaced with a new one.

D. Sample size calculation

Study one

The minimum sample size needed to develop a value set for EQ-5D-3L is 300 people [20] and for EQ-5D-5L is 1,000 people [21]. Normative data for any outcome instrument needs to be obtained from a sample that is an accurate representation of the general population [22]. The sample size needed to select a national representative sample of non-institutionalised Romanian adults aged 18 and older was estimated to be 1,613 people, with a maximum error of ±3% at a confidence level of 95%. Due to the complex nature of the survey, the sample size required for a simple random sample (SRS) was

For peer review only - http://bmjopen.bmj.com/site/about/guidelines.xhtml

adjusted by the design effect (deff) [23] and by a correction factor to account for the fact that only one person per household was selected (deff(p)). Given that neither the EQ-5D-3L or 5L has been used before to measure HRQoL in Romania, self-reported health, whereby respondents are asked to classify their current health status, was used as a proxy for HRQoL.

$$N = \frac{t^2 * p\,(1-p)}{\Delta^2} * deff * deff(p) = 896 * 1.5 * 1.2 = 1{,}613$$

t=1.96, given a 95% confidence interval; $\Delta = 0.03$;

p= 0.7 (70% of Romanians considered having good health status according to the 2015 National Survey on Quality of Life) [24];

deff=1.5 [25]; deff (p)=1.2

The expected non-response rate was set at 10% leading to a final sample size of 1,794 people.

Study two

Sample size calculations for study two were estimated using the same formula described above leading to a sample size of 606 people, with a maximum error of ±5% at a confidence level of 95%. The sample size was similarly inflated to cover a 10% non-response rate leading to a target sample size of 673 people.

IV. Pilot study

Study one

The study's brochure, informed consent forms and the software needed to collect the data was pretested with approximately 10 volunteers for wording, clarity, order of questions and potential technical problems. Additionally, the local team of investigators performed approximately 30 interviews in Bucharest to gain experience with performing valuation task interviews.

All interviewers attended a two days and half face-to-face training in October 2018. Prior to the training, interviewers were asked to read the interviewer's guideline and to familiarize themselves with the data collection tools prior to the face-to-face training. At the end of the training, all interviewers performed approximately five interviews in Bucharest with a convenience sample of respondents selected from hospitals and schools. The local team of investigators assessed the quality of these pilot interviews and provided personalized feedback to each interviewer.

Study two

The study's brochure and informed consent forms were pretested with a group of five volunteers from Dâmbovița county and four health mediators. The survey company also pretested the

For peer review only - http://bmjopen.bmj.com/site/about/guidelines.xhtml

contents of the survey for wording, order of questions and timings with approximately 10 volunteers from Cluj, a city in Romania. In collaboration with the local team of investigators, the survey company will provide an online training to all participating interviewers.

V. Data collection

Study one

Lay people will perform face-to-face interviews in respondents' homes using laptops connected to a secure online survey site (EQ-VT software version 2.1). Interviewers were selected from members of the national network of hospital assessors managed by the National Authority of Quality Management in Health from Romania and from members of patients' associations. In order to ease respondent recruitment, information about the study will be made available to the general public via social media and, where possible, more specific information will be given to the selected settlements via the Facebook page of the respective city hall (if available) or by contacting key community members.

A. Study questionnaire

The survey will consist of the following five block sections:

1. The EQ-5D-5L questionnaire and background questions on gender, age and experience of illness. EQ-5D-5L, one of the most widely used generic questionnaires to measure HRQoL [26], is a self-reported questionnaire that asks the respondent to assess his/her current health status on five dimensions (mobility, self-care, usual activities, pain/discomfort and anxiety/depression) and includes a visual analogue scale (EQ-VAS).
2. composite Time-Trade Off (cTTO) valuations tasks (EQ-5D-3L and 5L). In this section respondents will be asked to imagine living in an impaired health state for a defined period of time and indicate the years of remaining lifetime they will be willing to give up on to avoid living in the impaired health state. Respondents will complete two examples and three practice health states to get used to the task. Then they will assess ten EQ-5D-5L health states and three EQ-5D-3L health states each followed by a feedback module.
3. Discrete choice valuation (DCE) tasks (only for EQ-5D-5L). Respondents will be asked to state their preference between two hypothetical health states. They will value seven pairs of EQ-5D-5L health states followed by debriefing questions on the valuation tasks.
4. The EQ-5D-3L questionnaire. Respondents will be asked to fill in the EQ-5D-3L questionnaire. EQ-5D-3L measures health status using the same five dimensions as the 5L version, the only difference being that each dimension has only three levels (no problems", "some problems", and "extreme problems).

For peer review only - http://bmjopen.bmj.com/site/about/guidelines.xhtml

5. Country specific questionnaire. The survey will end with a series of sociodemographic questions on residence type, ethnicity, caregiver and parenting status, health literacy (one question that explores the respondent's level of confidence when interacting with healthcare professionals), preference over length or quality of life, marital status, education level, religion (affiliation, general religiosity, participation in religious services, praying), employment status and income.

B. Data quality control

The data quality control process will follow the recommendations of the most up-to-date version of the EQ-VT protocol [27 28]. An online Quality Control (QC) tool developed by the EuroQoL Foundation will be used to evaluate interviewers' performance. This tool determines protocol compliance, interviewers' effects and mean values by health state severity [29] and is used by the local team of investigators to provide personalized feedback via phone calls to all interviewers. Data quality control checks will be run after each interviewer will have performed a round of 10 interviews. Interviews are flagged as non-compliant if the explanations for the first two examples last for less than three minutes, if the worse than death (WTD) element is not shown in the examples, if the duration of cTTO tasks is less than five minutes, or if the value given to the worse health state is not the lowest and at least 0.5 higher than that of the state with the lowest value[29]. Poor performing interviewers will be re-trained and removed from the team if no improvement is seen after re-training. Checks on the "random-walk" implementation will also be performed. The local team of investigators will contact by phone a random selection of respondents (10%) to verify that interviews did take place.

Study two

Interviews will be conducted by professional staff from a survey company with experience in working with the Roma community. They will be face-to-face, pen and pencil interviews. Interviewers' access to communities will be facilitated by contacting leaders of the respective communities such as health mediators, social worker assistants or school mediators. All interviews will be conducted with care and sensitivity and respect for all participants' level of literacy. The study will be presented in simple words and if the participant can't write, the interviewer will sign the informed consent on his/her behalf if he/she desires to take part in the study.

To ease recruitment, just like for study one, information about the study will be made available to the general public via social media (Facebook page, project webpage) and, if possible, more specific information will be made available to the city halls or health mediators of the selected settlements.

A. Study questionnaire

The survey will consist of the following two sections:

1. The EQ-5D-5L questionnaire

For peer review only - http://bmjopen.bmj.com/site/about/guidelines.xhtml

2. Sociodemographic questionnaire. This will include the same questions as the Country specific questionnaire used in study one with the addition of two more questions: the availability of health mediators in the respective community and the ability of the respondent to read and write.

B. Data quality control

The data quality control process will be performed by the survey company in accordance with its internal procedures. Paper questionnaires will be validated on a rolling basis and incomplete questionnaires will undergo a "data recovery process" by re-contacting respondents and resuming unanswered items. Additionally, a random selection of 20% of respondents will be contacted by phone to verify that interviews did take place. If needed, field checks will also be performed.

VI. Analysis

Study one

The EQ-5D-3L value set will be developed using only cTTO data whereas the 5L value set will use both cTTO and DCE data. Different models, such as ordinary least squares (OLS), generalized least squares (GLS) and tobit, will be used to analyse cTTO data, whereas probit or logit models will be used to analyse the DCE data. A hybrid model that uses both cTTO and DCE data will also be developed for the EQ-5D-5L value set using the most innovative modelling techniques [30 31]. The model with the best fit will be chosen following theoretical considerations and comparisons of models based on the monotonic structure of the decrements, the standard errors, the ranking of the dimensions as well as the resulting predictions.

Respondents' responses to both EQ-5D-3L and 5L descriptive systems will be scored using the respective value sets and summary statistics (mean, standard deviation, median, 25th percentile and 75th percentile) by age groups and sex will be presented (population norms for the value set/utility index). The same summary statistics stratified by age groups and sex will also be presented for the EQ-VAS (i.e. population norms for EQ-VAS). The distribution of people and the odds ratios for reporting different levels of problems or any problem in all dimensions of the two EQ-5D questionnaires will be calculated and stratified by sociodemographic variables. The frequency of the most common health states in the general population in Romania will also be determined.

The effects of different variables, such as health literacy or religion, on health valuations will also be explored. This will be done using simple and multiple linear regression models that will have as dependent variable the TTO scores. Other explanatory variables considered for these models will be age, sex, place of residence (urban/rural), and educational level. All models will be adjusted for severity of health states with random effects to account for multiple valuations by the same respondent.

For peer review only - http://bmjopen.bmj.com/site/about/guidelines.xhtml

Study two

Potential differences in HRQoL according to sociodemographic characteristics and ethnicity will be explored in a subset of age and gender-matched sample of Roma population (study two) and general population (study one). If the data follows a normal distribution, t-tests and ANOVA analysis will be used to reflect differences across groups in HRQoL and for each dimension of the EQ-5D-5L questionnaire, otherwise non-parametric tests will be used. Multiple regression analysis will be used to study how the EQ-5D-5L visual analogue scale varies with sociodemographic characteristics, educational level, occupation, income, self-rated health and chronic diseases in the two populations.

For both study one and two, the survey sample distributions will be adjusted by applying weights based on 2011 census data to the age, sex and regional distribution of the two samples.

VII. Patient and Public involvement

Patients were not involved in the development of the research questions, selection of outcome measures and design of either Study one or two as the approach to address the research questions followed pre-established research methodology. Some of the trained interviewers that performed data collection in Study one are members of patients' associations in Romania. Suggestions from members of the general public (Study one) and from health mediators and Roma people from Dâmbovița county (Study two) were included in the design of the participant information sheets.

The results of this research will be communicated in two public workshops with local authorities and members of patients' associations and will also be available online at https://research.ncl.ac.uk/qolro/ and at http://www.romaniacurata.ro/valuemed/.

Discussion

Our study will estimate for the first time in Romania value sets and population norms for the EQ-5D questionnaires. This will constitute a stepping-stone to further development of HTA in Romania as it will potentially lead to more transparent and consistent decision-making in healthcare and to a more efficient use of relatively scarce local resources. It will also provide for the first time a measure of the health status of the general population in Romania and that of Roma communities. This could feed into better public health interventions and policies for either vulnerable or patient groups. Finally, if sample size targets are met, this could become the largest study conducted in Romania on HRQoL.

However, our study has a certain number of limitations. First of all, our respondents will be selected using "the birthday rule". "The birthday rule" is considered to be a quasi-probability procedure of selecting respondents [32], but it is the easiest and least time-consuming in terms of

For peer review only - http://bmjopen.bmj.com/site/about/guidelines.xhtml

interviewers' training and administration. It has also been implemented with good results in other representative surveys in Romania and elsewhere [33].

The survey weights that we will be using to correct the representativeness of the study's samples will be estimated based on the most recent census data available, more exactly the 2011 census data. Since 2011, migration rates have been increasing in Romania with the country currently having the highest growth in the size of its diaspora population after Syria [34]. Unfortunately, the 2011 census data has not yet been updated with the most recent data on the exact number of Romanian emigrants [35 36], so our weights might not accurately correct the representativeness of the two samples. Also, due to the current troubled political context in Romania, our interviewers might be faced in the field with a lower propensity of local residents to participate in surveys. This could lead to lower than predicted response rates for certain areas, such as urban areas, potentially affecting the quality of the data through non-response bias.

Finally, our survey on Roma communities will consist of self-identified Roma adults. Several studies in Romania have shown that self-identification and hetero-identification generally do not overlap [37 38]. Moreover, self-identified Roma tend to be more impoverished and with a higher residential segregation than hetero-identified Roma [37]. Hence, our comparison results might overestimate the differences between the two groups.

Ethics and dissemination

This study will be conducted in agreement with ethical principles set out in the Declaration of Helsinki and Regulation (EU) 679/2016 on the protection of natural persons with regard to the processing of personal data and the free movement of such data. Ethical approval was sought from the Bioethics Committee of Medicines and Medical Devices, Romania and from the Faculty of Medical Sciences Research Ethics Committee, part of Newcastle University's Research Ethics Committee, United Kingdom. As the project involves working with a vulnerable group, the project is of high risk in terms of ethics. Hence, all interviews will be conducted with care and sensitivity and with respect for participants' ethnicity, religion, language, sexual orientation or literacy level. All participants will be given enough time to read or be read the study's information brochure and to ask questions and discuss concerns regarding potential participation in the study. All the study's materials will have been pretested with volunteers and/or Roma mediators with the aim to try to ensure that information is simple and that words are easy to understand. Participants will be presented the study's participant information sheets, sign the informed consent forms, and be interviewed, all within one visit.

The dissemination plan for this project includes deliverables for the scientific community (scientific articles and conference presentations) and for the general public, such as press releases, brief communications on the project's website, Twitter and Facebook pages. Also, two public

For peer review only - http://bmjopen.bmj.com/site/about/guidelines.xhtml

workshops with local authorities, physicians and patients' associations will be organised to disseminate the results of the study to a broader audience. Finally, the anonymised datasets with EQ-5D-5L measures will be deposited in a centralised repository, such as Zenodo.

Bibliography

1. Spilker B. Quality of life assessments in clinical trials: New York: Raven Press 1990.
2. Torrance GW. Utility approach to measuring health-related quality of life. *J Chronic Dis* 1987;40(6):593-603. [published Online First: 1987/01/01]
3. Romero M, Vivas-Consuelo D, Alvis-Guzman N. Is Health Related Quality of Life (HRQoL) a valid indicator for health systems evaluation? *Springerplus* 2013;2:664. doi: 10.1186/2193-1801-2-664 [published Online First: 2013/12/20]
4. Cunningham SJ. Economic evaluation of healthcare--is it important to us? *Br Dent J* 2000;188(5):250-4. [published Online First: 2000/04/12]
5. Herdman M, Gudex C, Lloyd A, et al. Development and preliminary testing of the new five-level version of EQ-5D (EQ-5D-5L). *Qual Life Res* 2011;20(10):1727-36. doi: 10.1007/s11136-011-9903-x [published Online First: 2011/04/12]
6. EuroQol G. EuroQol--a new facility for the measurement of health-related quality of life. *Health Policy* 1990;16(3):199-208. [published Online First: 1990/11/05]
7. Boyle MH, Torrance GW. Developing multiattribute health indexes. *Med Care* 1984;22(11):1045-57. [published Online First: 1984/11/01]
8. Feeny D, Furlong W, Boyle M, et al. Multi-attribute health status classification systems. Health Utilities Index. *Pharmacoeconomics* 1995;7(6):490-502. doi: 10.2165/00019053-199507060-00004 [published Online First: 1995/05/08]
9. Brazier J, Roberts J, Deverill M. The estimation of a preference-based measure of health from the SF-36. *J Health Econ* 2002;21(2):271-92. [published Online First: 2002/04/10]
10. Wille N, Badia X, Bonsel G, et al. Development of the EQ-5D-Y: a child-friendly version of the EQ-5D. *Qual Life Res* 2010;19(6):875-86. doi: 10.1007/s11136-010-9648-y [published Online First: 2010/04/21]
11. Mogosan C, Stoica V, Mihai C, et al. Trends of rheumatoid arthritis monitorization in Romania. *J Med Life* 2010;3(3):330-7. [published Online First: 2010/10/16]
12. De Smedt D, Clays E, Doyle F, et al. Validity and reliability of three commonly used quality of life measures in a large European population of coronary heart disease patients. *Int J Cardiol* 2013;167(5):2294-9. doi: 10.1016/j.ijcard.2012.06.025 [published Online First: 2012/07/04]
13. De Smedt D, Clays E, Hofer S, et al. Validity and reliability of the HeartQoL questionnaire in a large sample of stable coronary patients: The EUROASPIRE IV Study of the European Society of Cardiology. *Eur J Prev Cardiol* 2016;23(7):714-21. doi: 10.1177/2047487315604837 [published Online First: 2015/09/12]
14. Paveliu M, Lorenzovici L, Tudose C. Oral anticoagulant treatment with antivitamin K in romania. A cost-effectiveness analysis of using INR home testing devices. *Farmacia* 2018;66(2):358 - 64.
15. Knies S, Evers SM, Candel MJ, et al. Utilities of the EQ-5D: transferable or not? *Pharmacoeconomics* 2009;27(9):767-79. doi: 10.2165/11314120-000000000-00000 [published Online First: 2009/09/18]
16. Eurostat. Healthcare expenditure statistics, 2017.
17. Precupetu I, Precupetu M. GINI Country Report: Growing Inequalities and their Impacts in Romania. GINI Country Reports: AIAS, Amsterdam Institute for Advanced Labour Studies, 2013.
18. Cook B, Wayne GF, Valentine A, et al. Revisiting the evidence on health and health care disparities among the Roma: a systematic review 2003-2012. *Int J Public Health* 2013;58(6):885-911. doi: 10.1007/s00038-013-0518-6 [published Online First: 2013/10/08]
19. Hoffmeyer-Zlotnik JH, Krebs D. Different methods of survey sampling in Germany. In: Ferligoj A, Kramberger A, eds. Developments in Data Analysis. Ljubljana: Metodoloski zvezki 1996.

For peer review only - http://bmjopen.bmj.com/site/about/guidelines.xhtml

20. Lamers LM, McDonnell J, Stalmeier PF, et al. The Dutch tariff: results and arguments for an effective design for national EQ-5D valuation studies. *Health economics* 2006;15(10):1121-32. doi: 10.1002/hec.1124
21. Oppe M, van Hout B. The "power" of eliciting EQ-5D-5L values. *EuroQoL Working Paper Series* 2017
22. Nolte MT, Shauver MJ, Chung KC. Analysis of four recruitment methods for obtaining normative data through a Web-based questionnaire: a pilot study. *Hand (N Y)* 2015;10(3):529-34. doi: 10.1007/s11552-014-9730-y
23. Laaksonen S. Sampling and estimation of multi-national surveys with examples from the European Social Survey, 2006.
24. National Institute of Statistics. Survey on quality of life. Romania. 2015
25. Rizzo C, Fabiani M, Amlot R, et al. Survey on the Likely Behavioural Changes of the General Public in Four European Countries During the 2009/2010 Pandemic. In: Manfredi P, D'Onofrio A, eds. Modeling the Interplay Between Human Behavior and the Spread of Infectious Diseases: Springer, New York, NY 2012.
26. Gusi N, Olivares PR, Rajendram R. The EQ-5D Health-Related Quality of Life Questionnaire. In: Preedy VR, Watson RR, eds. Handbook of Disease Burdens and Quality of Life Measures. New York, NY: Springer 2010.
27. Stolk EA, Ludwig K, Rand-Hendriksen K, et al. Overview, update and lessons learned from the international EQ-5D-5L valuation work: version 2 of the EQ-5D-5L valuation protocol *Value Health (submitted)*
28. Oppe M, Devlin NJ, van Hout B, et al. A program of methodological research to arrive at the new international EQ-5D-5L valuation protocol. *Value in health : the journal of the International Society for Pharmacoeconomics and Outcomes Research* 2014;17(4):445-53. doi: 10.1016/j.jval.2014.04.002
29. Ramos-Goni JM, Oppe M, Slaap B, et al. Quality Control Process for EQ-5D-5L Valuation Studies. *Value Health* 2017;20(3):466-73. doi: 10.1016/j.jval.2016.10.012
30. Ramos-Goni JM, Pinto-Prades JL, Oppe M, et al. Valuation and Modeling of EQ-5D-5L Health States Using a Hybrid Approach. *Med Care* 2017;55(7):e51-e58. doi: 10.1097/MLR.0000000000000283
31. Feng Y, Devlin NJ, Shah KK, et al. New methods for modelling EQ-5D-5L value sets: An application to English data. *Health Econ* 2018;27(1):23-38. doi: 10.1002/hec.3560 [published Online First: 2017/08/24]
32. Salmon CT, Nichols JS. The Next-Birthday Method of Respondent Selection. *Public Opin Quart* 1983;47(2):270-76. doi: Doi 10.1086/268785
33. Eurofund. 3rd European Quality of Life Survey: sampling report EU27 and non-EU countries: Eurofund, 2012.
34. United Nations DoEaSA, Population Division. International Migration Report 2015: Highlights (ST/ESA/SER.A/375), 2016.
35. Pisica S, Caragea N. Statistical overview of the international labor migration in Romania: Working papers 08, Ecological University of Bucharest, 2013.
36. Prada E, Ciupureanu C-A, Otoiu A. A Regional Analysis of Romanian Migration Determinants2015.
37. Ladányi J, Szelenyi I. The social construction of Roma ethnicity in Bulgaria, Romania and Hungary during market transition2001.
38. Cace S, Toader R, Vizireanu A. Romii din România. De la țap ispășitor la motor de dezvoltare. In: Gelu D, Ana I, eds.: Agentia Impreuna, 2013.

For peer review only - http://bmjopen.bmj.com/site/about/guidelines.xhtml

Acknowledgements

The authors would like to thank Kristina Ludwig and Elly Stolk from the EuroQoL Research Foundation for their comments on the protocol of the general population study (Study one) and support offered during the setup of study one. We would also like to thank Jan Deckers for his helpful comments and support in preparing the ethical submission for this project. We also thank our interviewers and especially the members of patients' associations in Romania for their help in performing the interviews of Study one.

Authors' contributions

The study was conceived by IGNA, EO, YO, LV, EB, and SP. EO wrote the first draft of the manuscript. All authors provided their intellectual input and approved the final manuscript.

Funding

These surveys are conducted by two teams of researchers that applied independently for funding. Given that several support activities are common across surveys, the two teams decided to offer each other methodological and scientific support. Hence, these surveys have several funding sources. The estimation of EQ-5D-5L value set, population norms and the comparison with Roma communities received funding from the European Union's Horizon 2020 research and innovation programme under the Marie Skłodowska-Curie grant agreement No 748612. It has also received funding from the EuroQol Research Foundation. The estimation of EQ-5D-3L value set and population norms received partial funding as part of a project run through the Romanian Operational Programme "Administrative Capacity" (POCA 2014-2020).

Competing interests

None.

Patient consent

Not required.

Ethics approval

The study protocol was reviewed and approved by the Bioethics Committee of Medicines and Medical Devices, Romania and by the Faculty of Medical Sciences Research Ethics Committee, part of Newcastle University's Research Ethics Committee, United Kingdom.

Data sharing statement

For peer review only

For peer review only - http://bmjopen.bmj.com/site/about/guidelines.xhtml

Not required at protocol stage.

For peer review only

For peer review only - http://bmjopen.bmj.com/site/about/guidelines.xhtml

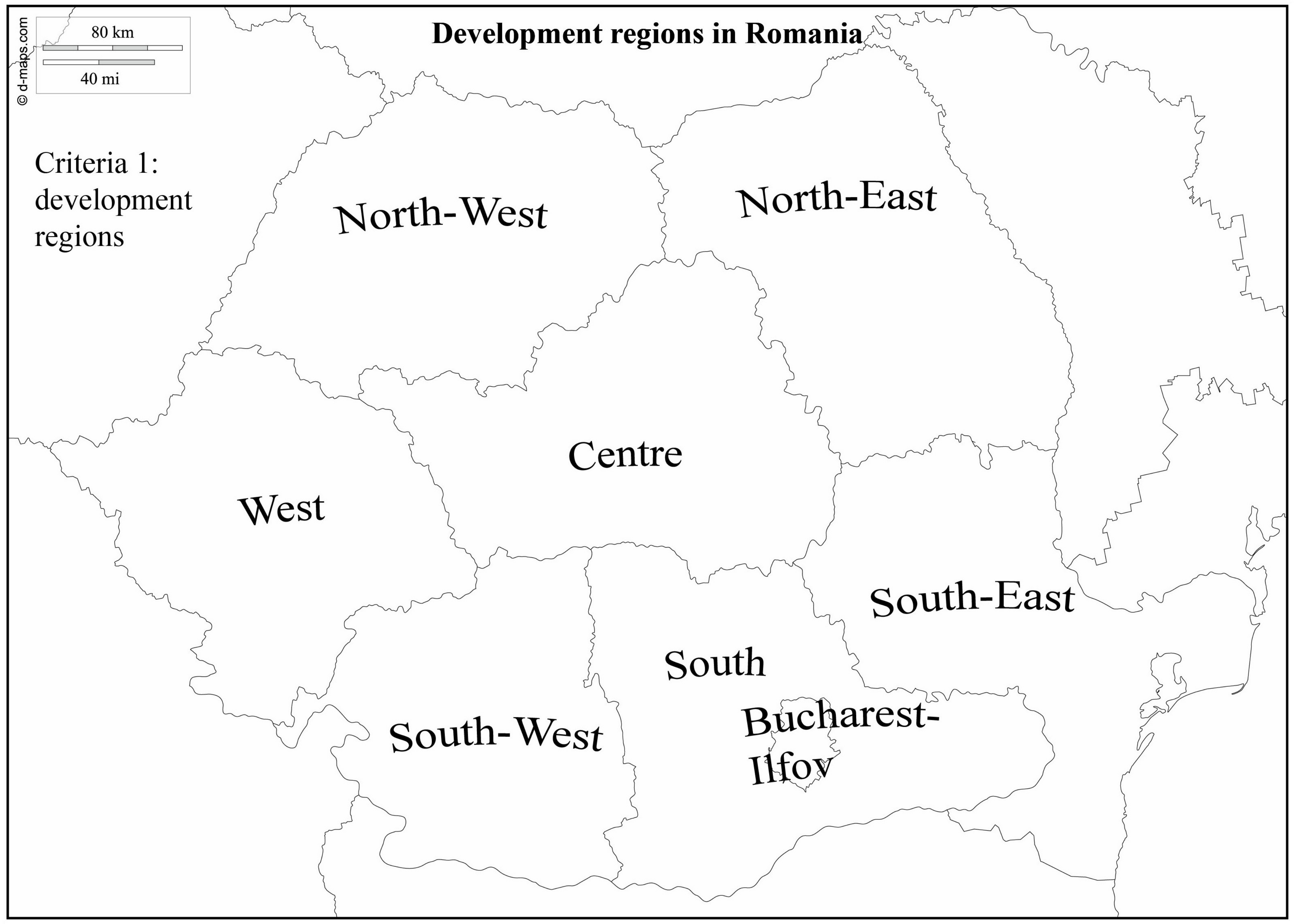

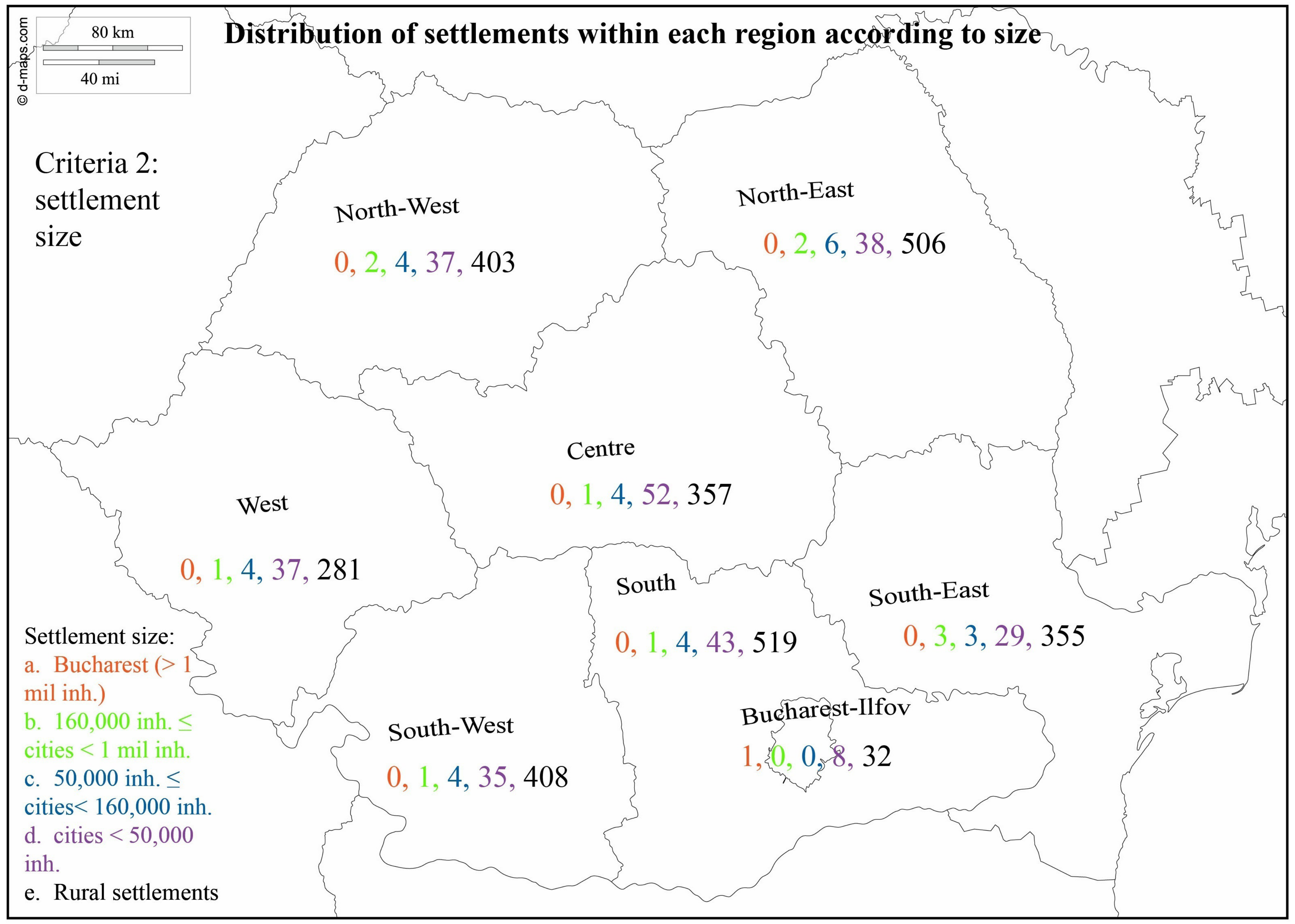

Criteria 1 + Criteria 2

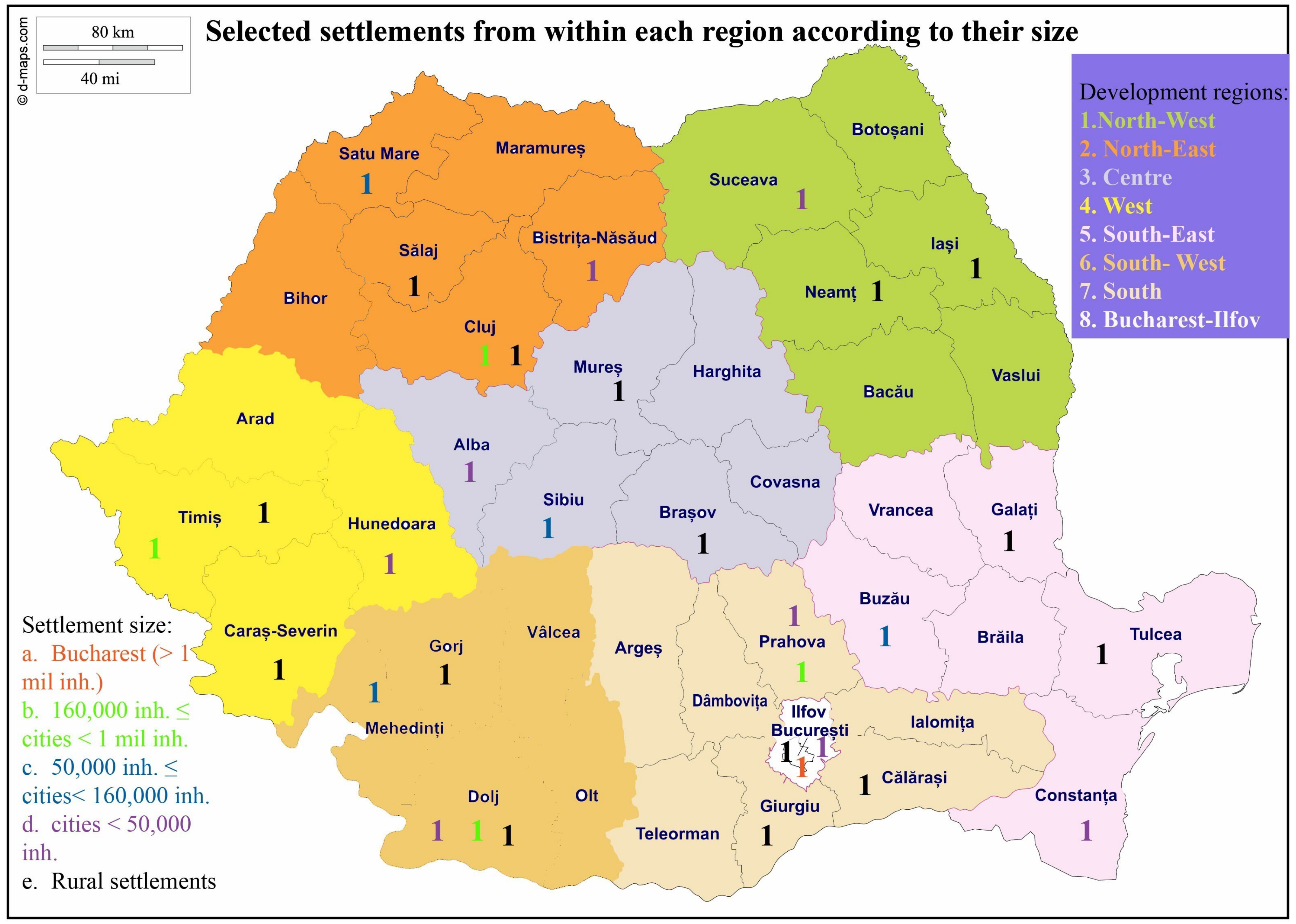

Original maps downloaded from https://d-maps.com/carte.php?num_car=5805&lang=en and https://d-maps.com/carte.php?num_car=25497&lang=en. Edited by EO.

BMJ Open

Measuring health-related quality of life in the general population and Roma communities in Romania: study protocol for two cross-sectional studies

Journal:	*BMJ Open*
Manuscript ID	bmjopen-2019-029067.R2
Article Type:	Protocol
Date Submitted by the Author:	18-Jun-2019
Complete List of Authors:	Olariu, Elena; Newcastle University, Institute of Health and Society<br>Paveliu, Sorin; Universitatea Titu Maiorescu Facultatea de Medicina, Department of Pharmacology and Pharmaeconomics<br>Baican, Eugen; Universitatea Babes-Bolyai, Department of Social Work<br>Oluboyede , Yemi; Newcastle University, Health Economics Group, Institute of Health and Society<br>Vale, Luke; Newcastle University, Health Economics Group, Institute of Health and Society<br>NICULESCU-ARON, ILEANA GABRIELA ; Academia de Studii Economice din Bucuresti, Department of Statistics and Econometrics, Faculty of Economic Cybernetics, Statistics and Informatics
**Primary Subject Heading**:	Health economics
Secondary Subject Heading:	Health policy
Keywords:	HEALTH ECONOMICS, Health policy < HEALTH SERVICES ADMINISTRATION & MANAGEMENT, Quality in health care < HEALTH SERVICES ADMINISTRATION & MANAGEMENT, PUBLIC HEALTH, STATISTICS & RESEARCH METHODS

SCHOLARONE™
Manuscripts

Measuring health-related quality of life in the general population and Roma communities in Romania: study protocol for two cross-sectional studies

Elena Olariu[1] , Marian-Sorin Paveliu[2]*, Eugen Baican[3], Yemi Oluboyede[1], Luke Vale[1], Ileana Gabriela Niculescu-Aron[4]

[1] Health Economics Group, Institute of Health and Society, Newcastle University, Newcastle upon Tyne, United Kingdom

[2] Department of Pharmacology and Pharmaeconomics, Faculty of General Medicine, Titu Maiorescu University, Bucharest, Romania

[3] Department of Social Work, Babes-Bolyai University, Cluj-Napoca, Romania

[4] Department of Statistics and Econometrics, Faculty of Economic Cybernetics, Statistics and Informatics, Bucharest University of Economic Studies, Bucharest, Romania

* Corresponding author

Address for correspondence: Marian-Sorin Paveliu MD, PhD, Faculty of General Medicine, Titu Maiorescu University, 67A Gheorghe Petrascu Str., district 3, Bucharest, code 031593, Romania. Email: sorinpaveliu@yahoo.com. Telephone: 0040726.200.627.

Word count: 3,968 words

For peer review only - http://bmjopen.bmj.com/site/about/guidelines.xhtml

For peer review only

Abstract

Introduction

The importance of health-related quality of life (HRQoL) is increasing and many healthcare authorities recommend the use of measures that account for both mortality and morbidity. This study will determine, for the first time in Romania, value sets for EQ-5D-3L and EQ-5D-5L questionnaires and their population norms (study one). It will also compare the HRQoL (measured with EQ-5D-5L) of Roma communities in Romania with that of the general population (study two).

Methods & analysis

Cross-sectional studies of face-to-face interviews conducted in representative samples of the Romanian general population and Romanian Roma communities. 1,614 non-institutionalized adults older than 18 will be interviewed using a computer-assisted interview for study one. Participants will complete EQ-5D-3L and 5L, 13 composite time-trade-off tasks (cTTO), seven discrete choice experiment questions (DCE) and sociodemographic questions. For study two, 606 non-institutionalized self-identified Roma people older than 18 will be interviewed using a pencil-and-paper interview. Participants will complete EQ-5D-5L and the same sociodemographic questions as for study one.

The 3L value set will be estimated using econometric models and the cTTO data. cTTO and DCE data will be used for the 5L value set. Population norms will be reported by age and gender. The odds ratios for reporting different levels of problems and the most common health states in the population will be estimated. For study two, t-tests and ANOVA will be used to explore differences between groups in HRQoL and for each EQ-5D dimension.

Ethics & dissemination

Ethics approval was given by the National Bioethics Committee of Medicines and Medical Devices Romania and Newcastle University's Research Ethics Committee.

Results will be published in peer-reviewed journals, presented at scientific conferences and on the project's website. The EQ-5D-5L anonymised datasets will be deposited in a centralised repository. Two public workshops with local authorities, physicians and patients' associations will be held.

Keywords: Health Economics, Health policy, Public Health, Statistics & Research Methods, Quality in healthcare

For peer review only - http://bmjopen.bmj.com/site/about/guidelines.xhtml

Article Summary

Strengths and limitations of this study

- First study to determine the value sets and population norms for both EQ-5D-3L and 5L for Romania.
- First study to determine HRQoL (as measured with EQ-5D-5L) of Roma population from Romania
- The representative samples include only persons who reside in the country at the time of the data collection. Romania has one of the highest migration rates in Europe: 17% of the total population of Romania (3.3 million of 19.6 million) are working abroad, many of them of Roma ethnicity.

For peer review only

For peer review only - http://bmjopen.bmj.com/site/about/guidelines.xhtml

Introduction

Health-Related Quality of Life (HRQoL) is a multidimensional concept that includes physical, psychological, functional, and social aspects [1] regarding a person's perception of the impact of health status on quality of life [2]..

In the last 20 years, the importance of HRQoL in health research has increased with many healthcare authorities recommending the use of indicators, such as quality-adjusted life years (QALYs), rather than relying on just indicators of mortality or morbidity alone [3]**.** QALYs are the mainstay of economic evaluations that help decision-makers determine how resources can be used to give the greatest benefits [4]. QALYs combine information on the length of life with information on quality of life, expressed as utilities. Several tools are available to measure individuals' health status and to attach utility values to these health states, such as the EQ-5D [5 6], Health Utilities Index (HUI) [7 8], Short Form 6-dimension (SF-6D)[9] and so on.

The EQ-5D is one of the most commonly used questionnaires to elicit health states utilities, having both a youth [10] and adult version. For general populations of adults two versions of the questionnaire have been developed, 3L and 5L, with the latter offering a more detailed description of health status [6]. In order to use the EQ-5D to estimate QALYs, a value set to reflect people's preferences for different health states is needed. At present, no value sets have ever been developed in Romania for either the 3L or 5L versions of the questionnaire. Researchers and decision makers have always used the value set from another jurisdiction, most commonly the value sets in the UK [11-14]. Using value sets from other countries might introduce bias as people's preferences can be culturally sensitive, differing from one country to another [15]. Therefore, local reimbursement and health technology assessment (HTA) decisions should be based on data applicable to the local context to avoid incorrect and inefficient decisions about the allocation of resources[16].

Besides their use in economic evaluations, tools such as the EQ-5D can be used to identify health problems in the community. The quality of life of patients or vulnerable groups can be compared with that of an average person from the population of interest with the help of population norms. This can help identify health inequalities between different groups. This in turn may help identify where healthcare and public health interventions are needed to reduce inequalities. This is of particular relevance to Romania where health inequalities have increased sharply in the past few years between regions and groups [17]. One particularly vulnerable group is represented by the Roma communities whose health status is consistently reported as being poorer than that of the general population [18]. Up to this date, the HRQoL of either the general population or Roma communities has never been determined with the EQ-5D questionnaire and no population norms exist in Romania for either of the two versions of the questionnaire.

For peer review only - http://bmjopen.bmj.com/site/about/guidelines.xhtml

The aim of this study is to determine the value sets and population norms for the EQ-5D questionnaires and to compare HRQoL (measured with EQ-5D-5L) between the general population and Roma communities.

Methods and analysis

I. Study design

A cross-sectional study design consisting of face-to-face interviews conducted in two national representative samples (general population and Roma communities) recruited from all regions of Romania will be conducted from November 2018 to April 2019.

II. Aim and objectives

Our survey aims to provide HRQoL data to support HTA and reimbursement decisions in Romania and to support the development of interventions that are more responsive to the needs of local communities in Romania. Our specific objectives are:

1. To develop a value set for EQ-5D-3L and 5L based on societal preferences in Romania (Study one, an Omnibus survey)
2. To determine the population norms of the Romanian version of EQ-5D-3L and 5L (Study one, an Omnibus survey)
3. To describe differences in HRQoL between Roma communities and the majority of the population according to sociodemographic characteristics, socioeconomic status and ethnicity (Study two)

III. Study population

A. Target population

Study one

Non-institutionalized adults older than 18 who reside in the country at the time of the data collection.

Study two

Non-institutionalized self-identified Roma adults older than 18 who reside in the country at the time of the data collection.

B. Sampling frame

Study one & Study two

The sampling frame consists of all polling stations in Romania (18,626 polling stations for 3,181 settlements) that are publicly available on the website of the Romanian Permanent Electoral Authority.

C. Sample design

For peer review only - http://bmjopen.bmj.com/site/about/guidelines.xhtml

Study one

The sample was selected using a three-stage probability sampling procedure stratified by region and settlement size. Romania's territory was divided into strata using two criteria built according to the recommendations of the National Institute of Statistics:

1. Regional divisions (development regions):
    a. North-East: Bacău, Botoşani, Iaşi, Neamţ, Suceava, Vaslui
    b. South-East: Brăila, Buzău, Constanţa, Galaţi, Tulcea, Vrancea
    c. South: Argeş, Călăraşi, Dâmboviţa, Giurgiu, Ialomiţa, Prahova, Teleorman
    d. South-West: Dolj, Gorj, Mehedinţi, Olt, Vâlcea
    e. West: Arad, Caraş-Severin, Hunedoara, Timiş
    f. North-West: Bihor, Bistriţa-Năsăud, Cluj, Maramureş, Satu Mare, Sălaj
    g. Centre: Alba, Braşov, Covasna, Harghita, Mureş, Sibiu
    h. Bucharest: Ilfov, Bucharest
2. Size of settlement:
    a. Bucharest (more than 1 million inhabitants)
    b. Cities with more than 160.000 inhabitants, but up to 1 million inhabitants (≤
    c. Cities with 50.000 – 160.000 inhabitants
    d. Cities with less than 50.000 inhabitants
    e. Rural settlements

The strata were obtained by crossing the categories of the two criteria described above: a total of 19 strata was obtained (see Table 1). The first three categories of the "size of settlement" criteria were not crossed with the criteria "regional divisions" given that the number of settlements in the resulting strata would have been too small.

Strata No.	***Strata label***	***Number of settlements (primary units) in the population***	***Number of settlements (primary units) in the sample***
1	Cities > 1 mil inh	1	1
2	160.000 inh. ≤ cities < 1 mil inh	11	4
3	50.000 inh. ≤ cities <160.000 inh.	29	4
4	South: cities < 50.000 inh.	43	1
5	South-East: cities < 50.000 inh	35	1
6	West: cities < 50.000 inh	37	1
7	Centre: cities < 50.000 inh	52	1

For peer review only - http://bmjopen.bmj.com/site/about/guidelines.xhtml

Strata No.	Strata label	Number of settlements (primary units) in the population	Number of settlements (primary units) in the sample
8	North-West: cities < 50.000 inh	37	1
9	North-East: cities < 50.000 inh	38	1
10	South-East: cities < 50.000 inh	29	1
11	Bucharest-Ilfov: cities < 50.000 inh	8	1
12	South-East: rural settlements	519	2
13	South: rural settlements	408	2
14	West: rural settlements	281	2
15	Centre: rural settlements	357	2
16	North-West: rural settlements	403	2
17	North-East: rural settlements	506	2
18	South-East: rural settlements	355	2
19	Bucharest-Ilfov: rural settlements	32	1
	**Total**	**3181**	**32**

Mil: million; inh: Inhabitants

Table 1 – Number of sampled primary units by development region and settlement size

Within each resulting stratum, settlements were selected using a simple random sampling procedure without replacement. A total of 32 settlements was selected (see Figure 1): one settlement from the first bracket of the second criteria, eight from the second and third brackets of the second criteria, eight from the fourth bracket of the second criteria (one city with less than 50,000 inhabitants from each development region) and 15 from the last bracket (two rural settlements for the first seven development regions and one rural settlement for Bucharest). The sample size for each strata was determined based on disproportionate allocation to strata to ensure that at least 30 respondents would be selected for each settlement.

……………………….. ……… Insert figure 1 about here……………………………

.Figure 1Geographical distribution of selected settlements according to development region and size

The second stage consists of selecting the polling stations that will be used as a starting point for the "random route" selection of participants. Polling stations were selected using a simple random sampling procedure without replacement for all settlements selected in the previous stage. The number of polling stations was determined so that a minimum of 20 people could be assigned to each polling station selected from a city and a minimum of 15 people to each polling station selected from a rural settlement. Hence, three polling stations were selected for cities with more than 50,000 inhabitants and two polling stations for cities with less than 50,000 inhabitants and rural settlements.

For peer review only - http://bmjopen.bmj.com/site/about/guidelines.xhtml

Given that rural settlements can consist of one or more villages, the two polling stations were selected, if needed, from two different villages. In case of Bucharest, two polling sections were selected for each of the six sectors of the city. In total 82 polling stations were selected.

The third stage consists of selecting households and individuals from each settlement and "voting district". This will be done using a random route sampling method [19]. Interviewers will perform a random walk per "voting district". The interviewer will begin his/her route on the street whose name comes first in alphabetical order from the list of streets that make up the voting district. Once the street has been identified, he/she will start with the household located at the lowest house number on the street. To select the next household, the interviewer will walk on the same side of the street and select the 3rd household in ascending order.

In each household, the respondent will be selected based on "birthday rule": the adult whose birthday comes next from the interviewer's visit will be interviewed. In case of household non-response, the interviewer will visit the household two more times on different days and at different hours. If no contact is established after three calls, then the household is replaced with a new one as per the procedure described above (third household selected).

Study two

In order to increase the comparability of the two samples (general population and Roma communities) study two used the same sampling design as study one. Roma participants were selected from the same settlements selected for study one. If, based on 2011 census data, no Roma communities were reported to be living in the selected settlements, these were replaced with other settlements from the same strata with higher Roma representation. Disproportionate allocation to strata was used to select the number of respondents from each stratum.

Households and individuals from each settlement will be selected either based on the random route technique ("birthday rule") in those settlements that match the ones selected for study one and on a snowball technique for the rest. In case of non-response, the interviewer will make two more attempts on different days and at different hours. If no contact is established after three calls, then the household is replaced with a new one.

D. Sample size calculation

Study one

The minimum sample size needed to develop a value set for EQ-5D-3L is 300 people [20] and for EQ-5D-5L is 1,000 people [21]. Normative data for any outcome instrument needs to be obtained from a sample that is an accurate representation of the general population [22]. The sample size needed to select a national representative sample of non-institutionalised Romanian adults aged 18 and older was estimated to be 1,613 people, with a maximum error of ±3% at a confidence level of 95%. Due to the complex nature of the survey, the sample size required for a simple random sample (SRS) was

For peer review only - http://bmjopen.bmj.com/site/about/guidelines.xhtml

adjusted by the design effect (deff) [23] and by a correction factor to account for the fact that only one person per household was selected (deff(p)). Given that neither the EQ-5D-3L or 5L has been used before to measure HRQoL in Romania, self-reported health, whereby respondents are asked to classify their current health status, was used as a proxy for HRQoL.

$$N = \frac{t^2 * p\,(1-p)}{\Delta^2} * deff * deff(p) = 896 * 1.5 * 1.2 = 1{,}613$$

t=1.96, given a 95% confidence interval; Δ = 0.03;

p= 0.7 (70% of Romanians considered having good health status according to the 2015 National Survey on Quality of Life) [24];

deff=1.5 [25]; deff (p)=1.2

The expected non-response rate was set at 10% leading to a final sample size of 1,794 people.

Study two

Sample size calculations for study two were estimated using the same formula described above leading to a sample size of 606 people, with a maximum error of ±5% at a confidence level of 95%. The sample size was similarly inflated to cover a 10% non-response rate leading to a target sample size of 673 people.

IV. Pilot study

Study one

The study's brochure, informed consent forms and the software needed to collect the data was pretested with approximately 10 volunteers for wording, clarity, order of questions and potential technical problems. Additionally, the local team of investigators performed approximately 30 interviews in Bucharest to gain experience with performing valuation task interviews.

All interviewers attended a two days and half face-to-face training in October 2018. Prior to the training, interviewers were asked to read the interviewer's guideline and to familiarize themselves with the data collection tools prior to the face-to-face training. At the end of the training, all interviewers performed approximately five interviews in Bucharest with a convenience sample of respondents selected from hospitals and schools. The local team of investigators assessed the quality of these pilot interviews and provided personalized feedback to each interviewer.

Study two

The study's brochure and informed consent forms were pretested with a group of five volunteers from Dâmbovița county and four health mediators. The survey company also pretested the

For peer review only - http://bmjopen.bmj.com/site/about/guidelines.xhtml

contents of the survey for wording, order of questions and timings with approximately 10 volunteers from Cluj, a city in Romania. In collaboration with the local team of investigators, the survey company will provide an online training to all participating interviewers.

V. Data collection

Study one

Lay people will perform face-to-face interviews in respondents' homes using laptops connected to a secure online survey site (EQ-VT software version 2.1). Interviewers were selected from members of the national network of hospital assessors managed by the National Authority of Quality Management in Health from Romania and from members of patients' associations. In order to ease respondent recruitment, information about the study will be made available to the general public via social media and, where possible, more specific information will be given to the selected settlements via the Facebook page of the respective city hall (if available) or by contacting key community members.

A. Study questionnaire

The survey consists of a computer-assisted interview that uses a version of the EQVT software that the EuroQoL foundation developed for the US EQ-5D-5L valuation study [26]. The interview will include the following five block sections:

1. The EQ-5D-5L questionnaire and background questions on gender, age and experience of illness. EQ-5D-5L, one of the most widely used generic questionnaires to measure HRQoL [27], is a self-reported questionnaire that asks the respondent to assess his/her current health status on five dimensions (mobility, self-care, usual activities, pain/discomfort and anxiety/depression) and includes a visual analogue scale (EQ-VAS).
2. composite Time-Trade Off (cTTO) valuations tasks (EQ-5D-3L and 5L). In this section respondents will be asked to imagine living in an impaired health state for a defined period of time and indicate the years of remaining lifetime they will be willing to give up on to avoid living in the impaired health state. Respondents will complete two examples and three practice health states to get used to the task. Then they will assess ten EQ-5D-5L health states and three EQ-5D-3L health states each followed by a feedback module.
3. Discrete choice valuation (DCE) tasks (only for EQ-5D-5L). Respondents will be asked to state their preference between two hypothetical health states. They will value seven pairs of EQ-5D-5L health states followed by debriefing questions on the valuation tasks.
4. The EQ-5D-3L questionnaire. Respondents will be asked to fill in the EQ-5D-3L questionnaire. EQ-5D-3L measures health status using the same five dimensions as the

For peer review only - http://bmjopen.bmj.com/site/about/guidelines.xhtml

5L version, the only difference being that each dimension has only three levels (no problems", "some problems", and "extreme problems).

5. Country specific questionnaire. The survey will end with a series of sociodemographic questions on residence type, ethnicity, caregiver and parenting status, health literacy (one question that explores the respondent's level of confidence when interacting with healthcare professionals), preference over length or quality of life, marital status, education level, religion (affiliation, general religiosity, participation in religious services, praying), employment status and income.

B. Data quality control

The data quality control process will follow the recommendations of the most up-to-date version of the EQ-VT protocol [28 29]. An online Quality Control (QC) tool developed by the EuroQoL Foundation will be used to evaluate interviewers' performance. This tool determines protocol compliance, interviewers' effects and mean values by health state severity [30] and is used by the local team of investigators to provide personalized feedback via phone calls to all interviewers. Data quality control checks will be run after each interviewer will have performed a round of 10 interviews. Interviews are flagged as non-compliant if the explanations for the first two examples last for less than three minutes, if the worse than death (WTD) element is not shown in the examples, if the duration of cTTO tasks is less than five minutes, or if the value given to the worse health state is not the lowest and at least 0.5 higher than that of the state with the lowest value[30]. Poor performing interviewers will be re-trained and removed from the team if no improvement is seen after re-training. Checks on the "random-walk" implementation will also be performed. The local team of investigators will contact by phone a random selection of respondents (10%) to verify that interviews did take place.

Study two

Interviews will be conducted by professional staff from a survey company with experience in working with the Roma community. They will be face-to-face, pen and pencil interviews. Interviewers' access to communities will be facilitated by contacting leaders of the respective communities such as health mediators, social worker assistants or school mediators. All interviews will be conducted with care and sensitivity and respect for all participants' level of literacy. The study will be presented in simple words and if the participant can't write, the interviewer will sign the informed consent on his/her behalf if he/she desires to take part in the study.

To ease recruitment, just like for study one, information about the study will be made available to the general public via social media (Facebook page, project webpage) and, if possible, more specific information will be made available to the city halls or health mediators of the selected settlements.

A. Study questionnaire

For peer review only - http://bmjopen.bmj.com/site/about/guidelines.xhtml

The survey will consist of the following two sections:

1. The EQ-5D-5L questionnaire. The EQ-5D-5L questionnaire was chosen due to its reduced ceiling effects [5] and better distributional parameters and substantial improvement in informativity [31].
2. Sociodemographic questionnaire. This will include the same questions as the Country specific questionnaire used in study one with the addition of two more questions: the availability of health mediators in the respective community and the ability of the respondent to read and write.

B. Data quality control

The data quality control process will be performed by the survey company in accordance with its internal procedures. Paper questionnaires will be validated on a rolling basis and incomplete questionnaires will undergo a "data recovery process" by re-contacting respondents and resuming unanswered items. Additionally, a random selection of 20% of respondents will be contacted by phone to verify that interviews did take place. If needed, field checks will also be performed.

VI. Analysis

Study one

The EQ-5D-3L value set will be developed using only cTTO data whereas the 5L value set will use both cTTO and DCE data. Different models, such as ordinary least squares (OLS), generalized least squares (GLS) and tobit, will be used to analyse cTTO data, whereas probit or logit models will be used to analyse the DCE data. A hybrid model that uses both cTTO and DCE data will also be developed for the EQ-5D-5L value set using the most innovative modelling techniques [32 33]. The model with the best fit will be chosen following theoretical considerations and comparisons of models based on the monotonic structure of the decrements, the standard errors, the ranking of the dimensions as well as the resulting predictions.

Respondents' responses to both EQ-5D-3L and 5L descriptive systems will be scored using the respective value sets and summary statistics (mean, standard deviation, median, 25th percentile and 75th percentile) by age groups and sex will be presented (population norms for the value set/utility index). The same summary statistics stratified by age groups and sex will also be presented for the EQ-VAS (i.e. population norms for EQ-VAS). The distribution of people and the odds ratios for reporting different levels of problems or any problem in all dimensions of the two EQ-5D questionnaires will be calculated and stratified by sociodemographic variables. The frequency of the most common health states in the general population in Romania will also be determined.

The effects of different variables, such as health literacy or religion, on health valuations will also be explored. This will be done using simple and multiple linear regression models that will have

For peer review only - http://bmjopen.bmj.com/site/about/guidelines.xhtml

as dependent variable the TTO scores. Other explanatory variables considered for these models will be age, sex, place of residence (urban/rural), and educational level. All models will be adjusted for severity of health states with random effects to account for multiple valuations by the same respondent.

Study two

Potential differences in HRQoL according to sociodemographic characteristics and ethnicity will be explored in a subset of age and gender-matched sample of Roma population (study two) and general population (study one). If the data follows a normal distribution, t-tests and ANOVA analysis will be used to reflect differences across groups in HRQoL and for each dimension of the EQ-5D-5L questionnaire, otherwise non-parametric tests will be used. Multiple regression analysis will be used to study how the EQ-5D-5L visual analogue scale varies with sociodemographic characteristics, educational level, occupation, income, self-rated health and chronic diseases in the two populations.

For both study one and two, the survey sample distributions will be adjusted by applying weights based on 2011 census data to the age, sex and regional distribution of the two samples.

VII. Patient and Public involvement

Patients were not involved in the development of the research questions, selection of outcome measures and design of either Study one or two as the approach to address the research questions followed pre-established research methodology. Some of the trained interviewers that performed data collection in Study one are members of patients' associations in Romania. Suggestions from members of the general public (Study one) and from health mediators and Roma people from Dâmbovița county (Study two) were included in the design of the participant information sheets..

The results of this research will be communicated in two public workshops with local authorities and members of patients' associations and will also be available online at https://research.ncl.ac.uk/qolro/ and at http://www.romaniacurata.ro/valuemed/.

Discussion

Our study will estimate for the first time in Romania value sets and population norms for the EQ-5D questionnaires. This will constitute a stepping-stone to further development of HTA in Romania as it will potentially lead to more transparent and consistent decision-making in healthcare and to a more efficient use of relatively scarce local resources. It will also provide for the first time a measure of the health status of the general population in Romania and that of Roma communities. This could feed into better public health interventions and policies for either vulnerable or patient groups. Finally, if sample size targets are met, this could become the largest study conducted in Romania on HRQoL.

For peer review only - http://bmjopen.bmj.com/site/about/guidelines.xhtml

For peer review only

However, our study has a certain number of limitations. First of all, our respondents will be selected using “the birthday rule”. “The birthday rule” is considered to be a quasi-probability procedure of selecting respondents [34], but it is the easiest and least time-consuming in terms of interviewers’ training and administration. It has also been implemented with good results in other representative surveys in Romania and elsewhere [35].

The survey weights that we will be using to correct the representativeness of the study’s samples will be estimated based on the most recent census data available, more exactly the 2011 census data. Since 2011, migration rates have been increasing in Romania with the country currently having the highest growth in the size of its diaspora population after Syria [36]. Unfortunately, the 2011 census data has not yet been updated with the most recent data on the exact number of Romanian emigrants [37 38], so our weights might not accurately correct the representativeness of the two samples. Also, due to the current troubled political context in Romania, our interviewers might be faced in the field with a lower propensity of local residents to participate in surveys. This could lead to lower than predicted response rates for certain areas, such as urban areas, potentially affecting the quality of the data through non-response bias.

Finally, our survey on Roma communities will consist of self-identified Roma adults. Several studies in Romania have shown that self-identification and hetero-identification generally do not overlap [39 40]. Moreover, self-identified Roma tend to be more impoverished and with a higher residential segregation than hetero-identified Roma [39]. Hence, our comparison results might overestimate the differences between the two groups.

Ethics and dissemination

This study will be conducted in agreement with ethical principles set out in the Declaration of Helsinki and Regulation (EU) 679/2016 on the protection of natural persons with regard to the processing of personal data and the free movement of such data. Ethical approval was sought from the Bioethics Committee of Medicines and Medical Devices, Romania and from the Faculty of Medical Sciences Research Ethics Committee, part of Newcastle University's Research Ethics Committee, United Kingdom. As the project involves working with a vulnerable group, the project is of high risk in terms of ethics. Hence, all interviews will be conducted with care and sensitivity and with respect for participants’ ethnicity, religion, language, sexual orientation or literacy level. All participants will be given enough time to read or be read the study’s information brochure and to ask questions and discuss concerns regarding potential participation in the study. All the study’s materials will have been pretested with volunteers and/or Roma mediators with the aim to try to ensure that information is simple and that words are easy to understand. Participants will be presented the study’s participant information sheets, sign the informed consent forms, and be interviewed, all within one visit.

For peer review only - http://bmjopen.bmj.com/site/about/guidelines.xhtml

The dissemination plan for this project includes deliverables for the scientific community (scientific articles and conference presentations) and for the general public, such as press releases, brief communications on the project's website, Twitter and Facebook pages. Also, two public workshops with local authorities, physicians and patients' associations will be organised to disseminate the results of the study to a broader audience. Finally, the anonymised datasets with EQ-5D-5L measures will be deposited in a centralised repository, such as Zenodo.

Bibliography

1. Spilker B. Quality of life assessments in clinical trials: New York: Raven Press 1990.
2. Torrance GW. Utility approach to measuring health-related quality of life. *J Chronic Dis* 1987;40(6):593-603. [published Online First: 1987/01/01]
3. Romero M, Vivas-Consuelo D, Alvis-Guzman N. Is Health Related Quality of Life (HRQoL) a valid indicator for health systems evaluation? *Springerplus* 2013;2:664. doi: 10.1186/2193-1801-2-664 [published Online First: 2013/12/20]
4. Cunningham SJ. Economic evaluation of healthcare--is it important to us? *Br Dent J* 2000;188(5):250-4. [published Online First: 2000/04/12]
5. Herdman M, Gudex C, Lloyd A, et al. Development and preliminary testing of the new five-level version of EQ-5D (EQ-5D-5L). *Qual Life Res* 2011;20(10):1727-36. doi: 10.1007/s11136-011-9903-x [published Online First: 2011/04/12]
6. EuroQol G. EuroQol--a new facility for the measurement of health-related quality of life. *Health Policy* 1990;16(3):199-208. [published Online First: 1990/11/05]
7. Boyle MH, Torrance GW. Developing multiattribute health indexes. *Med Care* 1984;22(11):1045-57. [published Online First: 1984/11/01]
8. Feeny D, Furlong W, Boyle M, et al. Multi-attribute health status classification systems. Health Utilities Index. *Pharmacoeconomics* 1995;7(6):490-502. doi: 10.2165/00019053-199507060-00004 [published Online First: 1995/05/08]
9. Brazier J, Roberts J, Deverill M. The estimation of a preference-based measure of health from the SF-36. *J Health Econ* 2002;21(2):271-92. [published Online First: 2002/04/10]
10. Wille N, Badia X, Bonsel G, et al. Development of the EQ-5D-Y: a child-friendly version of the EQ-5D. *Qual Life Res* 2010;19(6):875-86. doi: 10.1007/s11136-010-9648-y [published Online First: 2010/04/21]
11. Mogosan C, Stoica V, Mihai C, et al. Trends of rheumatoid arthritis monitorization in Romania. *J Med Life* 2010;3(3):330-7. [published Online First: 2010/10/16]
12. De Smedt D, Clays E, Doyle F, et al. Validity and reliability of three commonly used quality of life measures in a large European population of coronary heart disease patients. *Int J Cardiol* 2013;167(5):2294-9. doi: 10.1016/j.ijcard.2012.06.025 [published Online First: 2012/07/04]
13. De Smedt D, Clays E, Hofer S, et al. Validity and reliability of the HeartQoL questionnaire in a large sample of stable coronary patients: The EUROASPIRE IV Study of the European Society of Cardiology. *Eur J Prev Cardiol* 2016;23(7):714-21. doi: 10.1177/2047487315604837 [published Online First: 2015/09/12]
14. Paveliu M-S, Lorenzovici L, Tudose C. Oral anticoagulant treatment with antivitamin K in romania. A cost-effectiveness analysis of using INR home testing devices. *Farmacia* 2018;66(2):358 - 64.
15. Knies S, Evers SM, Candel MJ, et al. Utilities of the EQ-5D: transferable or not? *Pharmacoeconomics* 2009;27(9):767-79. doi: 10.2165/11314120-000000000-00000 [published Online First: 2009/09/18]
16. Eurostat. Healthcare expenditure statistics, 2017.
17. Precupetu I, Precupetu M. GINI Country Report: Growing Inequalities and their Impacts in Romania. GINI Country Reports: AIAS, Amsterdam Institute for Advanced Labour Studies, 2013.

For peer review only - http://bmjopen.bmj.com/site/about/guidelines.xhtml

18. Cook B, Wayne GF, Valentine A, et al. Revisiting the evidence on health and health care disparities among the Roma: a systematic review 2003-2012. *Int J Public Health* 2013;58(6):885-911. doi: 10.1007/s00038-013-0518-6 [published Online First: 2013/10/08]
19. Hoffmeyer-Zlotnik JH, Krebs D. Different methods of survey sampling in Germany. In: Ferligoj A, Kramberger A, eds. Developments in Data Analysis. Ljubljana: Metodoloski zvezki 1996.
20. Lamers LM, McDonnell J, Stalmeier PF, et al. The Dutch tariff: results and arguments for an effective design for national EQ-5D valuation studies. *Health economics* 2006;15(10):1121-32. doi: 10.1002/hec.1124
21. Oppe M, van Hout B. The "power" of eliciting EQ-5D-5L values. *EuroQoL Working Paper Series* 2017
22. Nolte MT, Shauver MJ, Chung KC. Analysis of four recruitment methods for obtaining normative data through a Web-based questionnaire: a pilot study. *Hand (N Y)* 2015;10(3):529-34. doi: 10.1007/s11552-014-9730-y
23. Laaksonen S. Sampling and estimation of multi-national surveys with examples from the European Social Survey, 2006.
24. National Institute of Statistics. Survey on quality of life. Romania. 2015
25. Rizzo C, Fabiani M, Amlot R, et al. Survey on the Likely Behavioural Changes of the General Public in Four European Countries During the 2009/2010 Pandemic. In: Manfredi P, D'Onofrio A, eds. Modeling the Interplay Between Human Behavior and the Spread of Infectious Diseases: Springer, New York, NY 2012.
26. Law EH, Pickard AS, Xie F, et al. Parallel Valuation: A Direct Comparison of EQ-5D-3L and EQ-5D-5L Societal Value Sets. *Med Decis Making* 2018;38(8):968-82. doi: 10.1177/0272989X18802797 [published Online First: 2018/11/08]
27. Gusi N, Olivares PR, Rajendram R. The EQ-5D Health-Related Quality of Life Questionnaire. In: Preedy VR, Watson RR, eds. Handbook of Disease Burdens and Quality of Life Measures. New York, NY: Springer 2010.
28. Stolk EA, Ludwig K, Rand-Hendriksen K, et al. Overview, update and lessons learned from the international EQ-5D-5L valuation work: version 2 of the EQ-5D-5L valuation protocol *Value Health (submitted)*
29. Oppe M, Devlin NJ, van Hout B, et al. A program of methodological research to arrive at the new international EQ-5D-5L valuation protocol. *Value in health : the journal of the International Society for Pharmacoeconomics and Outcomes Research* 2014;17(4):445-53. doi: 10.1016/j.jval.2014.04.002
30. Ramos-Goni JM, Oppe M, Slaap B, et al. Quality Control Process for EQ-5D-5L Valuation Studies. *Value Health* 2017;20(3):466-73. doi: 10.1016/j.jval.2016.10.012
31. Buchholz I, Janssen MF, Kohlmann T, et al. A Systematic Review of Studies Comparing the Measurement Properties of the Three-Level and Five-Level Versions of the EQ-5D. *Pharmacoeconomics* 2018;36(6):645-61. doi: 10.1007/s40273-018-0642-5 [published Online First: 2018/03/25]
32. Ramos-Goni JM, Pinto-Prades JL, Oppe M, et al. Valuation and Modeling of EQ-5D-5L Health States Using a Hybrid Approach. *Med Care* 2017;55(7):e51-e58. doi: 10.1097/MLR.0000000000000283
33. Feng Y, Devlin NJ, Shah KK, et al. New methods for modelling EQ-5D-5L value sets: An application to English data. *Health Econ* 2018;27(1):23-38. doi: 10.1002/hec.3560 [published Online First: 2017/08/24]
34. Salmon CT, Nichols JS. The Next-Birthday Method of Respondent Selection. *Public Opin Quart* 1983;47(2):270-76. doi: Doi 10.1086/268785
35. Eurofund. 3rd European Quality of Life Survey: sampling report EU27 and non-EU countries: Eurofund, 2012.
36. United Nations DoEaSA, Population Division. International Migration Report 2015: Highlights (ST/ESA/SER.A/375), 2016.
37. Pisica S, Caragea N. Statistical overview of the international labor migration in Romania: Working papers 08, Ecological University of Bucharest, 2013.
38. Prada E, Ciupureanu C-A, Otoiu A. A Regional Analysis of Romanian Migration Determinants2015.

For peer review only - http://bmjopen.bmj.com/site/about/guidelines.xhtml

39. Ladányi J, Szelenyi I. The social construction of Roma ethnicity in Bulgaria, Romania and Hungary during market transition2001.
40. Cace S, Toader R, Vizireanu A. Romii din România. De la țap ispășitor la motor de dezvoltare. In: Gelu D, Ana I, eds.: Agentia Impreuna, 2013.

Acknowledgements

The authors would like to thank Kristina Ludwig and Elly Stolk from the EuroQoL Research Foundation for their comments on the protocol of the general population study (Study one) and support offered during the setup of study one. We would also like to thank Jan Deckers for his helpful comments and support in preparing the ethical submission for this project. We also thank our interviewers and especially the members of patients' associations in Romania for their help in performing the interviews of Study one.

Authors' contributions

The study was conceived by IGNA, EO, YO, LV, EB, and SP. EO wrote the first draft of the manuscript. All authors provided their intellectual input and approved the final manuscript.

Funding

These surveys are conducted by two teams of researchers that applied independently for funding. Given that several support activities are common across surveys, the two teams decided to offer each other methodological and scientific support. Hence, these surveys have several funding sources. The estimation of EQ-5D-5L value set, population norms and the comparison with Roma communities received funding from the European Union's Horizon 2020 research and innovation programme under the Marie Skłodowska-Curie grant agreement No 748612. It has also received funding from the EuroQol Research Foundation. The estimation of EQ-5D-3L value set and population norms received partial funding as part of a project run through the Romanian Operational Programme "Administrative Capacity" (POCA 2014-2020).

Competing interests

None.

Patient consent

Not required.

Ethics approval

For peer review only - http://bmjopen.bmj.com/site/about/guidelines.xhtml

The study protocol was reviewed and approved by the Bioethics Committee of Medicines and Medical Devices, Romania and by the Faculty of Medical Sciences Research Ethics Committee, part of Newcastle University's Research Ethics Committee, United Kingdom.

Data sharing statement

Not required at protocol stage.

For peer review only

For peer review only - http://bmjopen.bmj.com/site/about/guidelines.xhtml

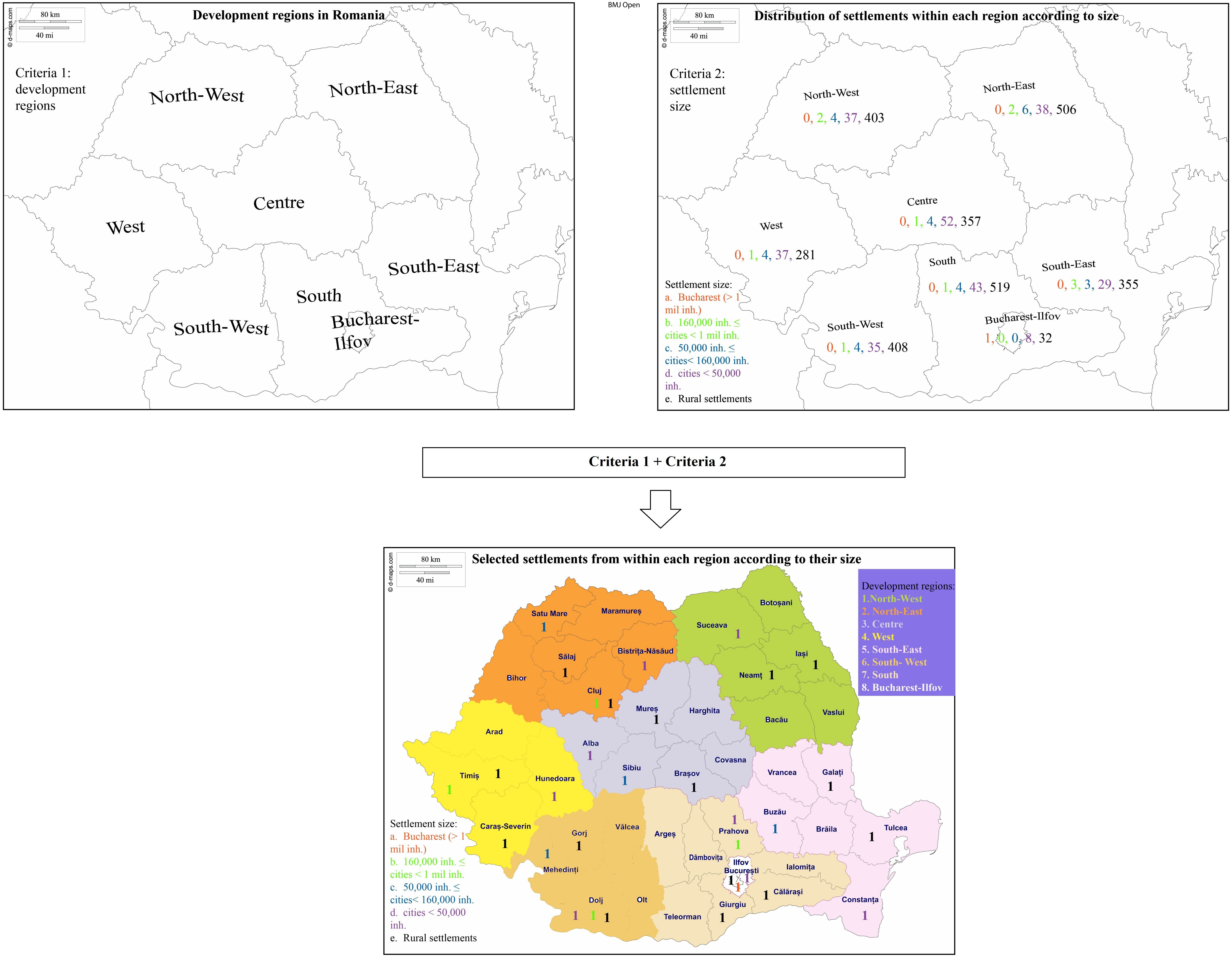

Original maps downloaded from https://d-maps.com/carte.php?num_car=5805&lang=en and https://d-maps.com/carte.php?num_car=25497&lang=en. Edited by EO.

For peer review only - http://bmjopen.bmj.com/site/about/guidelines.xhtml
